# Does functional system segregation mediate the effects of lifestyle on cognition in older adults?

**DOI:** 10.1016/j.neurobiolaging.2023.11.009

**Published:** 2024-02

**Authors:** Petar P. Raykov, Ethan Knights, Cam-CAN, Richard N. Henson

**Affiliations:** a Medical Research Council Cognition and Brain Sciences Unit, University of Cambridge, UK; b Department of Psychiatry, University of Cambridge, UK

**Keywords:** Fluid Intelligence, Memory, Functional System Segregation, Aging, Connectivity, FMRI

## Abstract

Healthy aging is typically accompanied by cognitive decline. Previous work has shown that engaging in multiple, non-work activities during midlife can have a protective effect on cognition several decades later, rendering it less dependent on brain structural health; the definition of “cognitive reserve”. Other work has shown that increasing age is associated with reduced segregation of large-scale brain functional networks. Here we tested the hypothesis that functional segregation (SyS) mediates this effect of middle-aged lifestyle on late-life cognition. We used fMRI data from three tasks in the CamCAN dataset, together with cognitive data on fluid intelligence, episodic memory, and retrospective lifestyle data from the Lifetime of Experiences Questionnaire (LEQ). In all three tasks, we showed that SyS related to fluid intelligence even after adjusting for the (nonlinear) age effects. However, we found no evidence that SyS in late-life mediated the relationship between non-specific (non-occupation) midlife activities and either measure of cognition in late-life. Thus, the brain correlates of cognitive reserve arising from mid-life activities remain to be discovered.

## Highlights

• System segregation (SyS) is stable across different brain states.
• SyS is negatively associated with age.
• Fluid intelligence, but not episodic memory, was robustly predicted by SyS.
• Late life SyS was not associated with midlife outside of work activities.

## 1 Introduction

Healthy aging is accompanied by decline in various cognitive functions (Gorbach et al., 2017, Nilsson et al., 2004, Nyberg et al., 2003, Tucker-Drob et al., 2019), with declines being most pronounced on tests of episodic memory and fluid intelligence. Studies using resting-state fMRI have also found age-related differences in the functional organisation of large-scale networks. Several such networks have been identified that show a modular organization, where regions from the same network tend to be functionally synchronised, but less synchronised with regions from other networks (Bullmore and Bassett, 2011, Bullmore and Sporns, 2009, Bullmore and Sporns, 2012, He and Evans, 2010, Heuvel and Sporns, 2013, Meunier et al., 2010). Crucially, this “functional system segregation” (SyS) - i.e., larger within-network connectivity compared to between-network connectivity – also seems to decline with age (Cao et al., 2014; Chan et al., 2014; Damoiseaux, 2017; Geerligs et al., 2014; Wig, 2017; see also Zonneveld et al., 2019). A modular functional network architecture may be important for optimal brain function and metabolic efficiency (Bullmore and Sporns, 2012, Van Den Heuvel and Sporns, 2011, Wig, 2017). As such, age-related reductions in SyS may have detrimental effects on cognition (Deery et al., 2023). Indeed, a study by Chan et al. (2014) found that lower SyS is associated with worse performance on an episodic memory task, while longitudinal studies found that a decrease in SyS is associated with a slowing of processing speed (Chong et al., 2019, Malagurski et al., 2020) and decrements in global cognition (Chan et al., 2021; Pedersen et al., 2021).

Although aging is on average associated with declining cognitive function, some people seem to be more resilient to this decline. Such individuals are said to have high “cognitive reserve” when they show better cognitive ability than would be expected from the deterioration of their brain structure, as consequence of aging, or diseases such as Alzheimer’s disease (Nilsson and Lövdén, 2018, Stern, 2012). It has been suggested that there are factors occurring earlier in people’s lives that increase their cognitive reserve later in life. For example, education and occupation have been shown to contribute positively to cognitive reserve (for review, see Richards and Deary, 2005). Recently, there has been increasing interest in how more modifiable lifestyle factors, such as leisure activities, can affect cognitive reserve. Gow and colleagues (2017) found that mid-life activities were associated with successful cognitive aging even after controlling for childhood cognitive ability, while Chan et al., 2018 found that mid-life activities outside the workplace correlated with late-life cognition over and above early-life education. Conversely, low-levels of physical and social activity in adulthood are risk factors for dementia (Livingston et al., 2020).

Although mid-life activities seem to be beneficial for cognitive reserve, it is still not clear through what brain mechanism they confer their benefits. Here we use data from the CamCAN cohort (www.cam-can.org) to address whether the positive effect of mid-life activities on late-life cognitive performance is mediated by SyS. In a previous report using the same cohort (Chan et al., 2018), we found that mid-life activities (outside occupation) moderated the relationship between measures of structural brain health and cognitive ability in later life, such that individuals who engaged in more leisure activities in their mid-life showed a reduced dependency of their fluid intelligence on their total gray matter volume, consistent with the concept of cognitive reserve. However, mid-life activities would be expected to affect the brain in some way, other than gray matter (Boyle et al., 2021, Husain, 2021). One obvious mechanism is functional connectivity, which has been proposed as a candidate correlate of cognitive reserve (Chan et al., 2018, Chan et al., 2021; Marques et al., 2016; Seeley et al., 2009; Stern, 2017; Zuo et al., 2020). For instance, Ewers et al. (2021) reported that higher SyS attenuated the relationship between Alzheimer’s disease severity and cognition, supporting ideas that SyS is a functional correlate of cognitive reserve (Marques et al., 2016, Stern, 2017, Steward et al., 2023). More specifically, given the prior relationships between SyS and cognitive performance cited above, mid-life activities could lead to better functional segregation later in life, which could compensate for age-related reductions in gray matter. If so, we would expect: 1) age to relate negatively to SyS, 2) SyS to relate positively to cognitive ability, even after adjusting for age, 3) mid-life activities to relate positively with SyS, and therefore 4) SyS to mediate the effect of mid-life activities on cognition.

We initially tested whether SyS decreases with age and independently predicts cognitive abilities (Predictions 1–2 above) across the full age-range in the CamCAN dataset (N = 627, aged 18–88). Two cognitive abilities were tested: episodic memory (as in Chan et al., 2014), through the WAIS logical memory task (Wechsler, 1991), and fluid intelligence (as in Chan et al., 2018), through the Cattell test (Cattell, 1971). Next, as in Chan et al., 2018, we tested Predictions 3–4 on a subset of N = 192 older participants (aged 66–88), who had provided retrospective reports of activities earlier in their life using the Lifetime of Experiences Questionnaire (LEQ) (Valenzuela and Sachdev, 2007). Additionally, in exploratory analyses, we tested 5) whether early-life specific LEQ activities, which mainly capture education, also related to late-life SyS measures (since Chan et al., 2018, also found a unique contribution of such early-life activities to late-life cognition), and 6) whether mid-life activities relate to current SyS, i.e., in mid-life rather than late-life participants. We estimated SyS from fMRI data recorded while the participants were in each of three states: i) resting with eyes closed, ii) watching a movie and iii) performing a simple sensory motor task (SMT).

## 2 Methods

### 2.1 Participants and Materials

We used data from the 627 (313 females, 314 males) adults, approximately uniformly distributed from 18 to 88 years of age, who had requisite data for each fMRI state and cognitive task from the Cambridge Centre for Aging and Neuroscience (Cam-CAN, www.cam-can.org, Shafto et al., 2014) cohort. We first focused on the full sample to examine whether we observe decreased functional segregation (SyS) over a wide age range. For our main hypotheses, examining the potential mediating role of SyS on the relationship between mid-life activities and cognition, we focused on a sub-sample of 192 older individuals (88 female and 104 male) aged 66–88 years. All participants scored 25 or higher on the mini mental state examination (Folstein et al., 1975), did not have a current diagnosis of dementia or mild cognitive impairment, and had normal or corrected to normal vision and hearing. Participants were native English speakers and had no neurological disorders (see Shafto et al., 2014, for further details).

All participants had completed measures of cognitive ability and had participated in a scanning session that included a resting-state scan, a sensory motor task and a movie task session. Two measures of cognitive ability were estimated: fluid intelligence and episodic memory. Fluid intelligence was measured using the Cattell Culture Fair test of fluid intelligence, which included 4 sub tests (Cattell, 1971). We took the first principal component over the four sub-tests to reduce the fluid intelligence measure into a single dimension. For episodic memory, we used the immediate, delayed recall and the recognition scores from the Wechsler Logical Memory task (Wechsler, 1991). To reduce the scores to a single measure per participant, we took the first principal component over the 3 memory scores.

We additionally had measures on participants’ lifestyle activities from the Lifetime of Experiences Questionnaire (LEQ, Valenzuela and Sachdev, 2007), modified for UK participants. The LEQ measures cognitively stimulating activities undertaken by participants during three life phases: youth (13–29 years), mid-life (30–64 years), and late-life (65 years onward). For each of these phases, activities are divided into “specific” ones, considered to be undertaken primarily during one phase (e.g., education for youth, occupation for mid-life), and “non-specific” activities, which apply to any life phase (e.g. socializing, playing sports). Young specific activities (YS - education), and Mid-life specific activities (MS - occupation) were scored based on UK’s National Career service categories and from standard occupational codes from the UK Office of National Statistics. Late-life specific activities (LS, or postretirement activities) reflected social and intellectual activities such as travel or participation in volunteer organizations. Here, our main focus was on mid-life non-specific activities (MA), which reflected engagement in 7 types of activities - social, intellectual and physical. There were 12 questions that addressed participation in (1) travel, (2) social outings, (3) playing a musical instrument, (4) artistic pastimes, (5) physical activity (mild, moderate, vigorous), (6) reading, and (7) speaking second language. The MA score was computed from the sum of all the questions.

The fMRI measures were acquired in the order: resting-state, sensorimotor task and movie. Each lasted around 8.5 min. During the resting scan, participants were asked to close their eyes and not think of anything in particular, but not to fall asleep. We note that a potential limitation of the study is that we did not measure whether participants fell asleep during the resting-state scan (Tagliazucchi and Laufs, 2014). If such sleep were more common in older people, this could explain some of the age effects in this state. In the sensory motor task, participants responded with a button press to simultaneous visual chequerboards and auditory tone presented randomly every few seconds. In the movie task, participants watched a shortened version of the Alfred Hitchcock movie “Bang! You’re Dead” (again, see Shafto et al., 2014, for further details).

### 2.2 MRI acquisition

All imaging data were collected on a Siemens Trio 3T MRI scanner with 32-channel head coil (more details can be found in Taylor et al., 2017). The rest and SMT tasks were acquired with a T2*-weighted echo planar imaging (EPI) sequence, resulting in 261 volumes, each containing 32 axial slices (acquired in descending order), with slice thickness of 3.7 mm and inter-slice gap of 20%. The repetition time (TR) was 1970 ms; echo time (TE) was 30 ms; flip angle was 78°; field of view was 192×192 mm and voxel size was 3×3x 4.44 mm. The movie task data were acquired with a similar EPI sequence, but with 5 TEs (of 9.4, 21.2, 33.0, 45.0, and 57.0 ms), a GRAPPA acceleration of 3, and a total of 193 volumes with TR = 2470 ms. The multi-echo data were combined by computing an average of the 5 echo times, weighted by their estimated T2* contrast. T1- and T2-weighted 1 mm isotropic structural MRI scans were also used (precise sequence parameters available here: https://camcan-archive.mrc-cbu.cam.ac.uk/dataaccess/pdfs/CAMCAN700_MR_params.pdf).

### 2.3 FMRI pre-processing

Data pre-processing was done in SPM 12 (http://www.fil.ion.ucl.ac.uk/spm), using the automatic analysis (AA) batching system (http://imaging.mrc-cbu.cam.ac.uk/imaging/AA). We used the same pre-processing strategy across the three states, as this has been shown to result in reliable functional connectivity matrices (Geerligs et al., 2015). Furthermore, recent work has suggested that task-induced functional connectivity may be more strongly associated with individual differences in cognition (Finn et al., 2017, Finn and Bandettini, 2021, Greene et al., 2018). Nonetheless, we also regressed out task-related activity from the SMT state (where the task is most obviously defined), and obtained almost identical SyS values, with a correlation of r = 0.995, p < 0.001 across participants, as without removing task-related effects. This suggests that our SyS values were not unduly influenced by common task-related activations relative to true functional connectivity.

To correct for image distortions due to field inhomogeneity, field maps were collected and applied to the fMRI data. Functional data were motion-corrected then slice-time corrected, and co-registered to the structural images. The high-resolution structural images were co-registered to the functional images, segmented and warped to a study-specific anatomical template using DARTEL procedures (Ashburner, 2007) and later affine-body registered to Montreal Neurological Institute (MNI) space. The segmented images were used to create white-matter (WM) and cerebrospinal fluid (CSF) masks for each participant by selecting only voxels with < 1% of gray matter and > 80% of WM/CSF. One voxel smoothing was applied to the functional data to avoid interpolation artefacts.

Additional pre-processing steps were applied to the functional data before computing the ROI connectivity matrices. Data were extracted from 500 ROIs taken from the Schaefer atlas (Schaefer et al., 2018). The ROIs were grouped into 17 resting state networks as defined by Yeo and colleagues (2011). For each ROI, we extracted the first temporal eigenvector (see Section 3.1.1 of Basti et al., 2020); though near-identical results were obtained using the straight average across voxels (SyS values from SVD versus from average correlated at r = 0.99 cross participants for the three states). We applied a high-pass filter removing frequencies lower than 0.01 Hz as this has been shown to result in more reliable connectivity estimates (Geerligs et al., 2017). The 6 motion parameters, mean signal from WM, CSF and global mean signal were included as covariates of no-interest. Additionally, for each of these nine confound regressors, we included their derivative, their square and their squared derivative (Ciric et al., 2017, Yan et al., 2013). We additionally accounted for excessive motion in the time-series by including a further set of regressors, each containing a single delta function for each volume during which head motion deviated more than 5 standard deviations from the root mean square of the detrended realignment parameters. Connectivity matrices were computed using Pearson correlation on the residual time-series. Correlation coefficients were Fisher transformed to Z statistics accounting for autocorrelation in the signal using the FSLnets package (Smith et al., 2011).

To compute system segregation (SyS), we used the networks defined by Yeo et al. (2011), but focused on ROIs that belonged to ‘associative’ networks. In other words, we excluded ‘sensory’ networks from our analyses, because SyS in associative networks has been more strongly linked to cognition (Chan et al., 2014; Pedersen et al., 2021). This left eleven Yeo networks: two Dorsal Attention sub Networks (DAN), two Ventral Attention Networks (VAN), three Frontoparietal Control Networks, three Default Mode Network (DMN), and a Temporal Parietal network (see Supplementary Fig. 1). In line with previous work, to avoid spurious negative associations introduced by global signal regression (GSR), we set negative correlations in the ROIxROI connectivity matrix to zero (Chan et al., 2014; Malagurski et al., 2020; Murphy and Fox, 2017). To compute SyS for each task, we followed the same procedure as Chan et al. (2014): we averaged within-network correlations (Z-statistics) and between-network correlations, then subtracted the average between-network correlation from the average within-network correlation, and normalized the result by dividing it by the average within-network correlation. This resulted in three SyS measures per participant, one for each task.

In Supplementary Materials, we explore whether the results reported here are stable when using different brain parcellations and network definitions. We compute the SyS measures with different resolutions of the Schaefer parcellation, and use the 7 and 17 Yeo networks. Conclusions from mediation models when focusing on SyS measures computed with 7 Yeo networks remained the same as the one reported in the main manuscript. Additionally, we compute SyS measures using the Craddock atlas with age-representative networks defined from the same data by Geerligs et al. (2015), and also the Gordon et al. (2016) atlas. Our results are stable across different atlases and networks.

### 2.4 Statistical analysis

All statistical analysis was done in R markdown (R Core Team, 2022, Xie et al., 2023). Initially, we examined whether SyS decreases with age across the whole Cam-CAN cohort, separately for each brain state. We predicted SyS using linear regression models including sex, a second-order polynomial expansion of age and their interactions. Afterwards, we examined the relationship between cognitive ability and SyS for each task, after accounting for sex and linear + quadratic effects of age and their interactions. Plots showing association between SyS and cognition were generated with the visreg package, which shows the association between a term of interest and the partial residuals (Breheny and Burchett, 2017). We additionally checked whether any observed effects were robust to “outliers” (influential observations) by examining whether the effects remained significant after fitting robust regression with the Huber loss function (Huber, 2011).

After examining the relationship between SyS and cognition over a wide age range, we focused on the subsample of older adults over 65. This allowed us to test whether SyS mediates the benefits in later life conferred by activities performed decades earlier in mid-life. As described in an OSF project (https://osf.io/bq3a7/wiki/home/), we first tested whether the SyS measure, from any task, was related to non-specific MA measures from the LEQ questionnaire, adjusting for sex, second-order effects of age and their interaction. We then examined whether any task-specific SyS measure mediated the relationship between MA and cognitive ability, using the “lavaan” R package (Rosseel, 2012).

Raw and processed fMRI images, along with AA pre-processing scripts, are available on https://camcan-archive.mrc-cbu.cam.ac.uk/dataaccess/. ROI-level connectivity matrices for each participant and brain state, as well as non-imaging data and all R code, are available on https://osf.io/bq3a7/.

## 3 Results

### 3.1 Confirmatory Analyses

These analyses are based on those stated in our OSF project https://osf.io/bq3a7/wiki/home/.

#### 3.1.1 Age predicts SyS across adult lifespan

For each functional task, we had SyS data from 627 adults. In Supplementary Figure 1, we show the 17 Yeo networks and Supplementary Figure 3 we visualise average functional connectivity matrices for a random selection of young (n = 79, aged 18–30) and a random selection of older (n = 79, aged 65–88) adults. Older groups tended to show weaker within-network connectivity on average, and some stronger between-network connectivity, as expected.

**Fig. 1:**
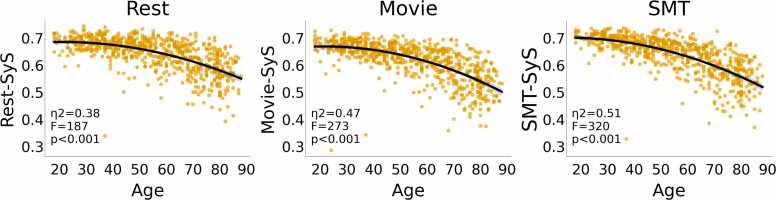
Predicting SyS from Age in full sample (N = 627). SyS decreases with age in each of the three brain states. Black line indicates second-order fit, and gray area demonstrates 95% bootstrapped intervals for the fit. Robust regression showed the age effects remained significant when accounting for possible “outlier” values.

Having extracted the SyS summary measure of functional segregation for each participant and brain state, a second-order polynomial regression against age showed significant linear and quadratic effects, whereby SyS showed an accelerating decrease with age in every state (see Figure 1 and Table 1). We did not observe significant effects of sex or sex-by-age interactions. The effect of age was driven by both decrease in within-network connectivity and increases in between-network connectivity (see Supplementary Figure 4).

**Table 1:** *Predicting SyS from Age in full sample (N = 627)*. Statistics from linear regression models predicting SyS from sex, a second-order polynomial expansion of age and their interactions. The terms age^1^ and age^2^ refer to the linear and quadratic effects of age respectively. Significant effects are shown in **bold**.

Task	Polynomial Term				
	age^1^	age^2^	Sex	age^1^:Sex	age^2^:Sex
***Rest***	**ß=-0.04**	**ß=0.01**	ß=0.00	ß=-0.00	ß=-0.00
***R***^***2***^=***0.37***	**T=-18.70**	**T**=**-4.84**	T=0.23	T=−0.39	T=−0.26
***df=621***	**p<0.001**	**p=0.001**	p=0.82	p=0.69	p=0.80
	**η**^**2**^=**0.36**	**η**^**2**^=**0.036**			
***Movie***	**ß=-0.05**	**ß=-0.01**	ß=0.00	ß=-0.00	ß=-0.00
***R***^***2***^=***0.46***	**T**=**-22.73**	**T** = **-5.42**	T=0.79	T=−0.19	T=−0.41
***df=621***	**p<0.001**	**p<0.001**	p=0.43	p=0.85	p=0.68
	**η**^**2**^**=0.45**	**η**^**2**^=**0.05**			
***SMT***	**ß=-0.05**	**ß=-0.01**	ß=-0.00	ß=0.00	ß=0.00
***R***^***2***^=***0.44***	**T**=**-24.73**	**T**=**-5.40**	T=0.45	T=0.00	T=1.05
***df=621***	**p<0.001**	**p=0.04**	p=0.65	p=1	p=0.30
	**η**^**2**^=**0.50**	**η**^**2**^=**0.04**			

#### 3.1.2 SyS predicts Cognition, above Age

**Fig. 2:**
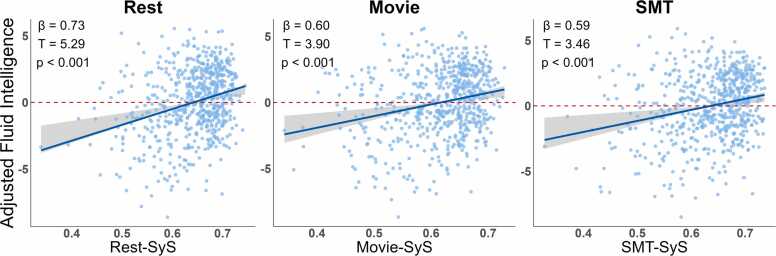
Predicting Fluid Intelligence from SyS in full sample (N = 627). Fluid intelligence was positively related to SyS in each of the three brain states, after adjusting for second-order effects of age (and sex, and interactions between all three variables). Scatter points show residuals after adjustment. Blue line indicates linear fit, and shaded area demonstrates 95% confidence intervals for the fit. Robust regression showed the relationships remained significant when accounting for possible “outlier” values.

Next, we examined whether SyS predicted cognitive performance, after controlling for sex, linear and quadratic effects of age, and allowing for all possible interactions between SyS, sex and age. We found that fluid intelligence was positively associated with SyS for each brain state (see Figure 2 and Table 2). We also found that the association between Fluid intelligence and SyS was dependent on the participant’s age and sex in the Rest task, such that older females showed a weaker association between SyS and intelligence (explored further in Supplementary Figure 5). We did not observe any other interactions in any of the three states.

**Table 2:** *Predicting Fluid Intelligence from SyS in full sample (N = 627)*. Statistics from linear models predicting Fluid Intelligence from SyS, after adjusting for sex, second-order effects of age and their interaction. See Table 1 legend for more details.

Task	Terms					
	SyS	SyS:age^1^	SyS:age^2^	SyS:Sex	Sex:age^1^	Sex:age^2^
***Rest***	**ß=0.73**	ß=-0.19	ß=-0.11	ß=-0.09	ß=-0.01	ß=-0.14
***R***^***2***^=***0.49***	**T=5.29**	T=-1.35	T=-0.96	T=-0.63	T=-0.08	T=-1.16
***df***=***615***	**p<0.001**	p=0.18	p=0.34	p=0.53	p=0.94	p=0.25
	**η**^**2**^=**0.04**					
***Movie***	**ß=0.6**	ß=-0.14	ß=-0.10	ß=-0.18	ß=-0.11	ß=-0.01
***R***^***2***^=***0.48***	**T**=**3.90**	T=-0.86	T=-0.87	T=-1.17	T=-0.67	T=-0.09
**df=615**	**p<0.001**	p=0.39	p=0.39	p=0.24	p=0.50	p=0.93
	**η**^**2**^=**0.03**					
***SMT***	**ß=0.59**	ß=0.07	ß=-0.24	ß=-0.24	ß=-0.14	ß=-0.10
***R***^***2***^=***0.48***	**T**=**3.46**	T=0.40	T=-1.93	T=-1.40	T=-0.83	T=-0.72
***df***=***615***	**p<0.001**	p=0.69	p=0.05	p=0.16	p=0.41	p=0.47
	**η**^**2**^=**0.03**					

Note: Three-way interactions between SyS, Sex and Linear or Quadratic effects of Age are not shown for space reasons.

**Fig. 3:**
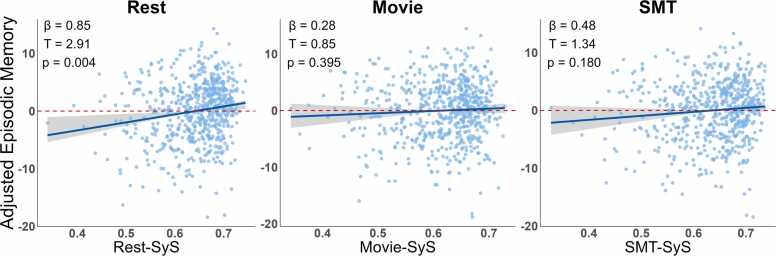
Predicting Episodic Memory from SyS in full sample (N = 627). Episodic memory was significantly related to SyS only in the Rest state.

For episodic memory, there was a significant relationship with SyS only in the Rest state, but not the Movie and SMT states (see Figure 3 and Table 3).

**Table 3:** *Predicting Episodic Memory from SyS in the full sample (N = 627)*. Statistics from linear models predicting Episodic Memory from SyS, after adjusting for sex, second-order effects of age and their interaction. See Table 1 legend for more details.

Task	Terms					
	SyS	SyS:age^1^	SyS:age^2^	SyS:Sex	Sex:age^1^	Sex:age^2^
***Rest***	**ß=0.85**	ß=-0.25	ß=-0.17	ß=-0.08	ß=-0.20	ß=0.25
***R***^***2***^=***0.16***	**T**=**2.91**	T=-0.79	T=-0.69	T=-0.29	T=-0.66	T=0.97
***df***=***615***	**p**=**0.003**	p=0.43	p=0.49	p=0.77	p=0.51	p=0.33
	**η**^**2**^=**0.01**					
***Movie***	ß=0.28	ß=-0.38	ß=-0.17	ß=-0.42	ß=-0.54	ß=0.35
***R***^***2***^=***0.16***	T=0.85	T=-1.11	T=-0.67	T=-1.29	T=-1.63	T=1.28
***df***=***615***	p=0.39	p=0.27	p=0.50	p=0.20	p=0.10	p=0.20
***SMT***	ß=0.48	ß=-0.29	ß=-0.21	ß=-0.42	ß=-0.51	ß=0.35
***R***^***2***^=***0.16***	T=1.34	T=-0.76	T=-0.81	T=-1.16	T=-1.38	T=1.16
***df***=***615***	p=0.18	p=0.45	p=0.42	p=0.25	p=0.17	p=0.25

Note: Three-way interactions between SyS, Sex and Linear or Quadratic effects of Age are not shown for space reasons.

Finally, in the Supplementary Materials, we show that the association between SyS and Fluid intelligence remains when adjusting for Education and Total Intracranial Volume (TIV) and is stronger than the association between SyS and episodic memory in all three states.

#### 3.1.3 Little evidence that mid-life activities relate to SyS in late-life

Having shown that SyS is related to age and cognition, at least across the whole adult sample used here, we next tested whether non-specific mid-life activities (MA) from the LEQ predicted SyS in late-life (since we previously showed that they do predict cognitive ability in late-life, Chan et al., 2018). For this, we focused on a sub-sample of 192 older (above 65 years) individuals for whom we had cognitive measures, LEQ scores and SyS measures from each of the three brain states. However, we observed a significant association between these mid-life activities and SyS only for the SMT state (Table 4). This was despite replicating the negative (linear) effect of Age on all SyS measures that we found in the full sample.

**Table 4:** Predicting SyS from MA in older adults (N = 192). Statistics from linear models predicting SyS from MA, after adjusting for second-order effects of age and sex.

Task	Terms			
	MA	age^1^	Sex	
***Rest***	ß=0.01	**ß**=**-0.020**	ß=-0.00	
***R***^***2***^=***0.08***	T=1.64	**T**=**-4.07**	T=-0.29	
***df=188***	p=0.10	**p<0.001**	p=0.77	
***Movie***	ß=0.01	**ß**=**-0.0240**	ß=-0.00	
***R***^***2***^=***0.12***	T=1.75	**T**=**-5.00**	T=-0.19	
***df***=***188***	p=0.08	**p<0.001**	p=0.85	
***SMT***	**ß**=**0.010**	**ß**=**-0.020**	ß=-0.00	
***R***^***2***^=***0.10***	**T**=**2.20**	**T**=**-4.24**	T=-0.32	
***df***=***188***	**p**=**0.029**	**p<0.001**	p=0.75	

#### 3.1.4 No evidence that SyS mediates effect of mid-life activities on late-life cognition

**Fig. 4:**
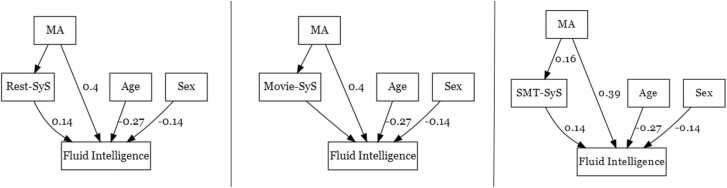
SyS from any state does not mediate the relationship between MA and Cattell in older adults (N = 192). Path diagram showing standardised coefficients that were significant in the mediation analysis in older adults using SyS, for each of the three states.

Given that there was little evidence that non-specific mid-life activities predicted SyS in late-life, it is unlikely that SyS mediates the effect of such MA on late-life fluid intelligence reported in Chan et al., 2018. Nonetheless, given this was our main, pre-registered hypothesis, we tested this mediation formally. We did not find significant mediation by any of the three SyS measures on fluid intelligence (see Figure 4), even for the SMT task where MA did relate to SyS beyond age. The percent of variance mediated, calculated as indirect effect / total effect was 4.2%, Z = 1.37, p = 0.17 for Rest; 3.7%, Z = 1.30, p = 0.19 for Movie and 5.4%, Z = 1.60, p = 0.11 for SMT. These results were replicated also when we computed SyS using different networks and atlases (see Supplementary Materials).

### 3.2 Exploratory Analyses

Given that we did not find evidence that mid-life non-specific activities affect SyS several decades later in late-life, we performed two exploratory analyses to see 1) whether late-life SyS is predicted by early-life specific activities (mainly education) instead, and 2) whether “current” mid-life SyS is predicted by mid-life non-specific activities in middle-aged participants.

#### 3.2.1 Little evidence that early-life activities (education) relate to late-life SyS

Given that LEQ scores specific to early-life – which capture education – also make an independent contribution to late-life cognition (Chan et al., 2018), we tested whether these predict SyS in late-life (even if mid-life LEQ scores did not). Again however, we found significant association between youth-specific LEQ activities and SyS only during the SMT task (ß = 0.010, t_(188)_ = 2.18, p = 0.031). No significant association was found for the Rest (ß = 0.007, t_(188)_ = 1.69, p = 0.09), and Movie (ß = 0.006, t_(188)_ = 1.24, p = 0.22) states after adjusting for (linear) age and sex.

#### 3.2.2 No evidence that mid-life activities relate to mid-life SyS

While we did not find evidence above that non-specific activities in mid-life relate to SyS in late-life, it is possible that such activities affect “current” SyS, i.e., during mid-life. Indeed, such a relationship was reported by Heneghan and colleagues (2022). We therefore ran exploratory analyses on n = 310 middle-aged (30 – 64 years) participants from whom we had LEQ scores. However, we again did not observe a significant relationship between mid-life non-specific LEQ activities and mid-life SyS during Rest (ß = 0.003, t_(306)_ = 1.50, p = 0.14), Movie (ß = 0.002, t_(305)_ = 0.79, p = 0.43) or SMT (ß = 0.001, t_(305)_ = 0.64, p = 0.53), after adjusting for (linear) age and sex.

#### 3.2.3 Pre-processing Options

Finally, we wanted to examine whether the SyS measure is sensitive to various pre-processing options, so compared the effects of these options on the relationship between SyS and cognition. The results are described in Supplementary Materials, but in brief, the positive relationship between SyS and fluid intelligence remained significant even when 1) not including global signal regression (GSR) and 2) regressing out mean connectivity instead (Geerligs et al., 2017), showing that it is robust to these major choices. However, it is worth noting that the relationship was no longer significant when 1) not thresholding to exclude negative connections (without GSR), or 2) when using partial correlation to estimate “direct” connections instead.

## 4 Discussion

We replicated previous studies by Chan et al. (2014) and Pedersen et al. (2021), in finding that functional system segregation (SyS) decreases across the adult lifespan, with an accelerated decrease in older adults, and is positively associated with cognitive performance, even after controlling for such age effects. More specifically, we replicated the previously observed relationship between resting-state SyS and episodic memory, after adjusting for linear and quadratic effects of age. However, we did not observe significant association between episodic memory and SyS in the other functional states. Extending previous studies, we found that the relationship between SyS and fluid intelligence was even stronger than that between SyS and memory, and was present regardless of whether SyS was measured during rest (as in previous studies), or during a stimulating movie, or during a simple sensorimotor task.

However, we found no support for our a priori (pre-registered) hypothesis that the effect of mid-life activities on late-life was mediated through SyS. This hypothesis was based on our finding that mid-life activities unrelated to occupation (as measured by the retrospective LEQ) were positively associated with fluid intelligence several decades later, and reduced the dependency of late-life fluid intelligence on brain structure Chan et al., 2018). However, we found little evidence that late-life SyS was positively associated with mid-life activities (except perhaps in the sensorimotor task). More importantly, we found no evidence for that SyS mediates the effect of mid-life activities on late-life cognition.

In exploratory analyses, we also failed to find evidence (except in the sensorimotor task) for a relationship between late-life SyS and activities specific to early life (which is mainly education in the LEQ), despite our finding of an independent contribution of such early-life activities to fluid intelligence in late-life (D. Chan et al., 2018). However, we note that a recent longitudinal study found that having a college degree can have a protective effect on rate of decline of SyS, which has predictive value for changes in dementia severity (Chan et al., 2021). This highlights the need for longitudinal study of brain network re-organization. Finally, we also failed to find evidence for a relationship between mid-life activities and “current” SyS in mid-life.

Thus, the late-life functional brain correlates of such mid-life activities remains unknown. Note that this does not mean that SyS is not relevant to cognitive reserve (e.g., in attenuating the relationship between Alzheimer’s disease severity and cognition; Chan et al., 2021; Marques et al., 2016; Stern, 2017); our results suggest only that mid-life activities exert their contribution to cognitive reserve via mechanisms not related to SyS. In other words, it is likely that multiple factors contribute to cognitive reserve, of which mid-life activity is just one, and each factor may be mediated by different brain correlates. An alternative brain mediator of mid-life activities on late-life cognition might be, for example, white-matter integrity (which may also be related to SyS, but only partially; Pedersen et al., 2021); something that can be investigated in future studies. Another possible biological mechanism might be BDNF-induced synaptogenesis and neurogenesis, which have been found to moderate the relationship between cognition and brain health (Buchman et al., 2016, Ward et al., 2015).

Finally, we note our study had several limitations. For one, the cross-sectional nature of the data prevented us from testing how longitudinal changes in SyS may be related to lifestyle activities and cognition (e.g., Chan et al., 2021). Secondly, the LEQ scores did not allow us to separate different types of activities, such as physical, social or intellectual (Borgeest et al., 2020), and these could have affected cognition and SyS differently. Thirdly, we focused on global changes in functional connectivity; however, recent work suggests that modifiable life factors may affect functional connectivity between specific brain regions (Fjell et al., 2016, Morris et al., 2021, Zonneveld et al., 2019). Fourthly, it is difficult to ascertain whether we fully controlled for head motion, which is known to bias functional connectivity estimates from fMRI (and head motion is known to increase with age, e.g., Geerligs et al., 2017; Savalia et al., 2017).

Another issue to keep in mind is the wide range of different options available for estimating SyS from fMRI, in terms of ROI and network definition, and the estimation of functional connectivity. We explore some of these options in the Supplementary Materials. For example, we show that the relationship between SyS and fluid intelligence was relatively robust to whether or not we performed global signal regression; a pre-processing option that has been contentious in the literature (see Aquino et al., 2020; Murphy and Fox, 2017; Saad et al., 2012). The relationship was also robust to whether we regressed out mean functional connectivity instead, which we previously proposed as a way to adjust for fMRI confounds like vascular effects of aging (Geerligs et al., 2017). We also showed that the relationship was reasonably robust to the ROI definition (whether we used Schaefer, Craddock or Gordon atlases), to the number of ROIs and networks (at least with Schaefer ROIs), and to whether or not we excluded non-associative networks. However, it should be noted that it was important for the SyS-intelligence relationship to exclude negative connections, and that the use of partial correlation to more closely estimate direct connections removed any significant relationship between SyS and fluid intelligence. We hope these explorations will be useful to future studies of fMRI system segregation, and help resolve any difficulties in replicating its relationship to age, cognition, or any other factor such as lifestyle choice.

In conclusion, we show that SyS shows accelerating decreases with age and is positively associated with fluid intelligence, even after removing such effects of age. However, we could not find effects of mid-life (or early-life) activities on late-life SyS, as a potential functional brain correlate of cognitive reserve. We suggest that future work examine other functional or structural brain measures that might mediate the effects of lifestyle on late-life cognition.

## *Cam-CAN corporate author

Lorraine K Tyler, Carol Brayne, Edward T Bullmore, Andrew C Calder, Rhodri Cusack, Tim Dalgleish, John Duncan, Richard N Henson, Fiona E Matthews, William D Marslen-Wilson, James B Rowe, Meredith A Shafto; Karen Campbell, Teresa Cheung, Simon Davis, Linda Geerligs, Rogier Kievit, Anna McCarrey, Abdur Mustafa, Darren Price, David Samu, Jason R Taylor, Matthias Treder, Kamen A Tsvetanov, Janna van Belle, Nitin Williams, Daniel Mitchell, Simon Fisher, Else Eising, Ethan Knights; Lauren Bates, Tina Emery, Sharon Erzinçlioglu, Andrew Gadie, Sofia Gerbase, Stanimira Georgieva, Claire Hanley, Beth Parkin, David Troy; Tibor Auer, Marta Correia, Lu Gao, Emma Green, Rafael Henriques; Jodie Allen, Gillian Amery, Liana Amunts, Anne Barcroft, Amanda Castle, Cheryl Dias, Jonathan Dowrick, Melissa Fair, Hayley Fisher, Anna Goulding, Adarsh Grewal, Geoff Hale, Andrew Hilton, Frances Johnson, Patricia Johnston, Thea Kavanagh-Williamson, Magdalena Kwasniewska, Alison McMinn, Kim Norman, Jessica Penrose, Fiona Roby, Diane Rowland, John Sargeant, Maggie Squire, Beth Stevens, Aldabra Stoddart, Cheryl Stone, Tracy Thompson, Ozlem Yazlik, Dan Barnes, Marie Dixon, Jaya Hillman, Joanne Mitchell, Laura Villis.

## Funding

Cam-CAN was supported by the 10.13039/501100000268Biotechnology and Biological Sciences Research Council Grant BB/H008217/1. R.N.H. and P.R. were supported by the UK Medical Research Council [SUAG/046/G101400]. E.K. was supported by the European Union Horizon 2020 Research and Innovation Program (LifeBrain) Grant Agreement 732592.

## CRediT authorship contribution statement

**Petar P. Raykov**: Study design, Analysis, Interpretation, Writing Draft. **Ethan Knights**: Study design, Conceptualization, Analysis. **Cam-CAN**: Data collection and verification. **Rik N. Henson:** Study design, Conceptualization, Editing, Reviewing. All authors read and approved the final manuscript.

## Declaration of Competing Interest

The authors declare no competing financial interests.

## References

[bib1] Aquino K.M., Fulcher B.D., Parkes L., Sabaroedin K., Fornito A. (2020). Identifying and removing widespread signal deflections from fMRI data: Rethinking the global signal regression problem. Neuroimage.

[bib2] Ashburner J. (2007). A fast diffeomorphic image registration algorithm. Neuroimage.

[bib3] Basti A., Nili H., Hauk O., Marzetti L., Henson R.N. (2020). Multi-dimensional connectivity: a conceptual and mathematical review. NeuroImage.

[bib4] Borgeest G.S., Henson R.N., Shafto M., Samu D., Cam-CAN, Kievit R.A. (2020). Greater lifestyle engagement is associated with better age-adjusted cognitive abilities. Plos One.

[bib5] Boyle P.A., Wang T., Yu L., Wilson R.S., Dawe R., Arfanakis K., Schneider J.A., Bennett D.A. (2021). To what degree is late life cognitive decline driven by age-related neuropathologies?. Brain.

[bib6] Breheny P., Burchett W. (2017). Visualization of regression models using visreg. R. J..

[bib7] Buchman A.S., Yu L., Boyle P.A., Schneider J.A., De Jager P.L., Bennett D.A. (2016). Higher brain BDNF gene expression is associated with slower cognitive decline in older adults. Neurology.

[bib8] Bullmore E., Bassett D.S. (2011). Brain graphs: graphical models of the human brain connectome. Annu. Rev. Clin. Psychol..

[bib9] Bullmore E., Sporns O. (2009). Complex brain networks: graph theoretical analysis of structural and functional systems. Nat. Rev. Neurosci..

[bib10] Bullmore E., Sporns O. (2012). The economy of brain network organization. Nat. Rev. Neurosci..

[bib11] Cao M., Wang J.-H., Dai Z.-J., Cao X.-Y., Jiang L.-L., Fan F.-M., Song X.-W., Xia M.-R., Shu N., Dong Q. (2014). Topological organization of the human brain functional connectome across the lifespan. Dev. Cogn. Neurosci..

[bib12] Cattell R.B. (1971). In *Abilities: their structure, growth, and action.* (pp. xxii, 583–xxii, 583).

[bib13] Chan D., Shafto M., Kievit R., Matthews F., Spink M., Valenzuela M., Henson R.N. (2018). Lifestyle activities in mid-life contribute to cognitive reserve in late-life, independent of education, occupation, and late-life activities. Neurobiol. Aging.

[bib14] Chan M.Y., Park D.C., Savalia N.K., Petersen S.E., Wig G.S. (2014). Decreased segregation of brain systems across the healthy adult lifespan. Proc. Natl. Acad. Sci..

[bib15] Chan M.Y., Na J., Agres P.F., Savalia N.K., Park D.C., Wig G.S. (2018). Socioeconomic status moderates age-related differences in the brain’s functional network organization and anatomy across the adult lifespan. Proc. Natl. Acad. Sci..

[bib16] Chan M.Y., Han L., Carreno C.A., Zhang Z., Rodriguez R.M., LaRose M., Hassenstab J., Wig G.S. (2021). Long-term prognosis and educational determinants of brain network decline in older adult individuals. Nat. Aging.

[bib17] Chong J.S.X., Ng K.K., Tandi J., Wang C., Poh J.-H., Lo J.C., Chee M.W.L., Zhou J.H. (2019). Longitudinal changes in the cerebral cortex functional organization of healthy elderly. J. Neurosci..

[bib18] Ciric R., Wolf D.H., Power J.D., Roalf D.R., Baum G.L., Ruparel K., Shinohara R.T., Elliott M.A., Eickhoff S.B., Davatzikos C. (2017). Benchmarking of participant-level confound regression strategies for the control of motion artifact in studies of functional connectivity. Neuroimage.

[bib19] Damoiseaux J.S. (2017). Effects of aging on functional and structural brain connectivity. Neuroimage.

[bib20] Deery H.A., Di Paolo R., Moran C., Egan G.F., Jamadar S.D. (2023). The older adult brain is less modular, more integrated, and less efficient at rest: a systematic review of large‐scale resting‐state functional brain networks in aging. Psychophysiology.

[bib21] Ewers M., Luan Y., Frontzkowski L., Neitzel J., Rubinski A., Dichgans M., Hassenstab J., Gordon B.A., Chhatwal J.P., Levin J. (2021). Segregation of functional networks is associated with cognitive resilience in Alzheimer’s disease. Brain.

[bib22] Finn E.S., Bandettini P.A. (2021). Movie-watching outperforms rest for functional connectivity-based prediction of behavior. NeuroImage.

[bib23] Finn E.S., Scheinost D., Finn D.M., Shen X., Papademetris X., Constable R.T. (2017). Can brain state be manipulated to emphasize individual differences in functional connectivity?. Neuroimage.

[bib24] Fjell A.M., Sneve M.H., Storsve A.B., Grydeland H., Yendiki A., Walhovd K.B. (2016). Brain events underlying episodic memory changes in aging: a longitudinal investigation of structural and functional connectivity. Cereb. Cortex.

[bib25] Folstein M.F., Folstein S.E., McHugh P.R. (1975). “Mini-mental state”: a practical method for grading the cognitive state of patients for the clinician. J. Psychiatr. Res..

[bib26] Geerligs L., Maurits N.M., Renken R.J., Lorist M.M. (2014). Reduced specificity of functional connectivity in the aging brain during task performance. Hum. Brain Mapp..

[bib27] Geerligs L., Rubinov M., Henson R.N. (2015). State and trait components of functional connectivity: individual differences vary with mental state. J. Neurosci..

[bib28] Geerligs L., Tsvetanov K.A., Henson R.N. (2017). Challenges in measuring individual differences in functional connectivity using fMRI: the case of healthy aging. Hum. Brain Mapp..

[bib29] Gorbach T., Pudas S., Lundquist A., Orädd G., Josefsson M., Salami A., de Luna X., Nyberg L. (2017). Longitudinal association between hippocampus atrophy and episodic-memory decline. Neurobiol. Aging.

[bib30] Gordon E.M., Laumann T.O., Adeyemo B., Huckins J.F., Kelley W.M., Petersen S.E. (2016). Generation and evaluation of a cortical area parcellation from resting-state correlations. Cereb. Cortex.

[bib31] Gow A.J., Pattie A., Deary I.J. (2017). Lifecourse activity participation from early, mid, and later adulthood as determinants of cognitive aging: the lothian birth cohort 1921. J. Gerontol.: Ser. B.

[bib32] Greene A.S., Gao S., Scheinost D., Constable R.T. (2018). Task-induced brain state manipulation improves prediction of individual traits. Nat. Commun..

[bib33] He Y., Evans A. (2010). Graph theoretical modeling of brain connectivity. Curr. Opin. Neurol..

[bib34] Heneghan A., Deng F., Wells K., Ritchie K., Muniz-Terrera G., Ritchie C.W., Lawlor B., Naci L. (2022). Modifiable lifestyle activities affect cognition in cognitively healthy middle-aged individuals at risk for late-life Alzheimer’s Disease. J. Alzheimer’s Dis., *Prepr.*.

[bib35] Heuvel M.P. Van Den, Sporns O. (2013). Network hubs in the human brain. Trends Cogn. Sci..

[bib36] Huber P.J. (2011). In International encyclopedia of statistical science.

[bib37] Husain M. (2021). Speak, memory: on cognitive reserve and brain resilience. Brain.

[bib38] Livingston G., Huntley J., Sommerlad A., Ames D., Ballard C., Banerjee S., Brayne C., Burns A., Cohen-Mansfield J., Cooper C., Costafreda S.G., Dias A., Fox N., Gitlin L.N., Howard R., Kales H.C., Kivimäki M., Larson E.B., Ogunniyi A., Mukadam N. (2020). Dementia prevention, intervention, and care: 2020 report of the <em>Lancet</em> Commission. Lancet.

[bib39] Malagurski B., Liem F., Oschwald J., Mérillat S., Jäncke L. (2020). Functional dedifferentiation of associative resting state networks in older adults–a longitudinal study. Neuroimage.

[bib40] Marques P., Moreira P., Magalhães R., Costa P., Santos N., Zihl J., Soares J., Sousa N. (2016). The functional connectome of cognitive reserve. Hum. Brain Mapp..

[bib41] Meunier D., Lambiotte R., Bullmore E.T. (2010). Modular and hierarchically modular organization of brain networks. Front. Neurosci..

[bib42] Morris T.P., Chaddock-Heyman L., Ai M., Anteraper S.A., Castañon A.N., Whitfield-Gabrieli S., Hillman C.H., McAuley E., Kramer A.F. (2021). Enriching activities during childhood are associated with variations in functional connectivity patterns later in life. Neurobiol. Aging.

[bib43] Murphy K., Fox M.D. (2017). Towards a consensus regarding global signal regression for resting state functional connectivity MRI. NeuroImage.

[bib44] Nilsson J., Lövdén M. (2018). Naming is not explaining: future directions for the “cognitive reserve” and “brain maintenance” theories. Alzheimer’s Res. Ther..

[bib45] Nilsson L.-G., Adolfsson R., Bäckman L., de Frias C.M., Molander B., Nyberg L. (2004). Betula: a prospective cohort study on memory, health and aging. Aging Neuropsychol. Cogn..

[bib46] Nyberg L., Maitland S.B., Rönnlund M., Bäckman L., Dixon R.A., Wahlin Å., Nilsson L.-G. (2003). Selective adult age differences in an age-invariant multifactor model of declarative memory. Psychol. Aging.

[bib47] Pedersen R., Geerligs L., Andersson M., Gorbach T., Avelar-Pereira B., Wåhlin A., Rieckmann A., Nyberg L., Salami A. (2021). When functional blurring becomes deleterious: reduced system segregation is associated with less white matter integrity and cognitive decline in aging. Neuroimage.

[bib48] R Core Team. (2022). R: A Language and Environment for Statistical Computing. https://www.r-project.org/.

[bib49] Richards M., Deary I.J. (2005). A life course approach to cognitive reserve: a model for cognitive aging and development?. Annl. Neurol. Off. J. Am. Neurol. Assoc. Child Neurol. Soc..

[bib50] Rosseel Y. (2012). lavaan: An R package for structural equation modeling. J. Stat. Softw..

[bib51] Saad Z.S., Gotts S.J., Murphy K., Chen G., Jo H.J., Martin A., Cox R.W. (2012). Trouble at rest: how correlation patterns and group differences become distorted after global signal regression. Brain Connect..

[bib52] Savalia N.K., Agres P.F., Chan M.Y., Feczko E.J., Kennedy K.M., Wig G.S. (2017). Motion‐related artifacts in structural brain images revealed with independent estimates of in‐scanner head motion. Hum. Brain Mapp..

[bib53] Schaefer A., Kong R., Gordon E.M., Laumann T.O., Zuo X.-N., Holmes A.J., Eickhoff S.B., Yeo B.T.T. (2018). Local-global parcellation of the human cerebral cortex from intrinsic functional connectivity MRI. Cereb. Cortex.

[bib54] Seeley W.W., Crawford R.K., Zhou J., Miller B.L., Greicius M.D. (2009). Neurodegenerative diseases target large-scale human brain networks. Neuron.

[bib55] Shafto M.A., Tyler L.K., Dixon M., Taylor J.R., Rowe J.B., Cusack R., Calder A.J., Marslen-Wilson W.D., Duncan J., Dalgleish T. (2014). The Cambridge Centre for Ageing and Neuroscience (Cam-CAN) study protocol: a cross-sectional, lifespan, multidisciplinary examination of healthy cognitive ageing. BMC Neurol..

[bib56] Smith S.M., Miller K.L., Salimi-Khorshidi G., Webster M., Beckmann C.F., Nichols T.E., Ramsey J.D., Woolrich M.W. (2011). Network modelling methods for FMRI. NeuroImage.

[bib57] Stern Y. (2012). Cognitive reserve in ageing and Alzheimer’s disease. Lancet Neurol..

[bib58] Stern Y. (2017). An approach to studying the neural correlates of reserve. Brain Imaging Behav..

[bib59] Steward A., Biel D., Brendel M., Dewenter A., Roemer S., Rubinski A., Luan Y., Dichgans M., Ewers M., Franzmeier N. (2023). Functional network segregation is associated with attenuated tau spreading in Alzheimer’s disease. Alzheimer’s Dement..

[bib60] Tagliazucchi E., Laufs H. (2014). Decoding wakefulness levels from typical fMRI resting-state data reveals reliable drifts between wakefulness and sleep. Neuron.

[bib61] Taylor J.R., Williams N., Cusack R., Auer T., Shafto M.A., Dixon M., Tyler L.K., Cam-CAN, Henson R.N. (2017). The Cambridge Centre for Ageing and Neuroscience (Cam-CAN) data repository: structural and functional MRI, MEG, and cognitive data from a cross-sectional adult lifespan sample. NeuroImage.

[bib62] Thomas Yeo B.T., Krienen F.M., Sepulcre J., Sabuncu M.R., Lashkari D., Hollinshead M., Roffman J.L., Smoller J.W., Zöllei L., Polimeni J.R. (2011). The organization of the human cerebral cortex estimated by intrinsic functional connectivity. J. Neurophysiol..

[bib63] Tucker-Drob E.M., Brandmaier A.M., Lindenberger U. (2019). Coupled cognitive changes in adulthood: a meta-analysis. Psychol. Bull..

[bib64] Valenzuela M.J., Sachdev P. (2007). Assessment of complex mental activity across the lifespan: development of the Lifetime of Experiences Questionnaire (LEQ). Psychol. Med..

[bib65] Van Den Heuvel M.P., Sporns O. (2011). Rich-club organization of the human connectome. J. Neurosci..

[bib66] Ward D.D., Summers M.J., Saunders N.L., Ritchie K., Summers J.J., Vickers J.C. (2015). The BDNF Val66Met polymorphism moderates the relationship between cognitive reserve and executive function. Transl. Psychiatry.

[bib67] Wechsler C. (1991).

[bib68] Wig G.S. (2017). Segregated systems of human brain networks. Trends Cogn. Sci..

[bib69] Xie, Y., Allaire, J., & Horner, J. (2023). markdown: Render Markdown with commonmark. https://github.com/rstudio/markdown.

[bib70] Yan C.-G., Craddock R.C., Zuo X.-N., Zang Y.-F., Milham M.P. (2013). Standardizing the intrinsic brain: Towards robust measurement of inter-individual variation in 1000 functional connectomes. NeuroImage.

[bib71] Zonneveld H.I., Pruim R.H.R., Bos D., Vrooman H.A., Muetzel R.L., Hofman A., Rombouts S.A.R.B., van der Lugt A., Niessen W.J., Ikram M.A. (2019). Patterns of functional connectivity in an aging population: the rotterdam study. Neuroimage.

[bib72] Zuo N., Salami A., Liu H., Yang Z., Jiang T. (2020). Functional maintenance in the multiple demand network characterizes superior fluid intelligence in aging. Neurobiol. Aging.

