# Supplementary material for: Does functional system segregation mediate the effects of lifestyle on cognition in older adults?
Source: Neurobiol Aging. 2024 Feb;134:126–34. doi: 10.1016/j.neurobiolaging.2023.11.009 (PMC10789480; doi:10.1016/j.neurobiolaging.2023.11.009)
Supplement: Supplementary file 1 — Supplementary material [file mmc1.pdf]

Supplementary Materials for Raykov, Knights, & Henson

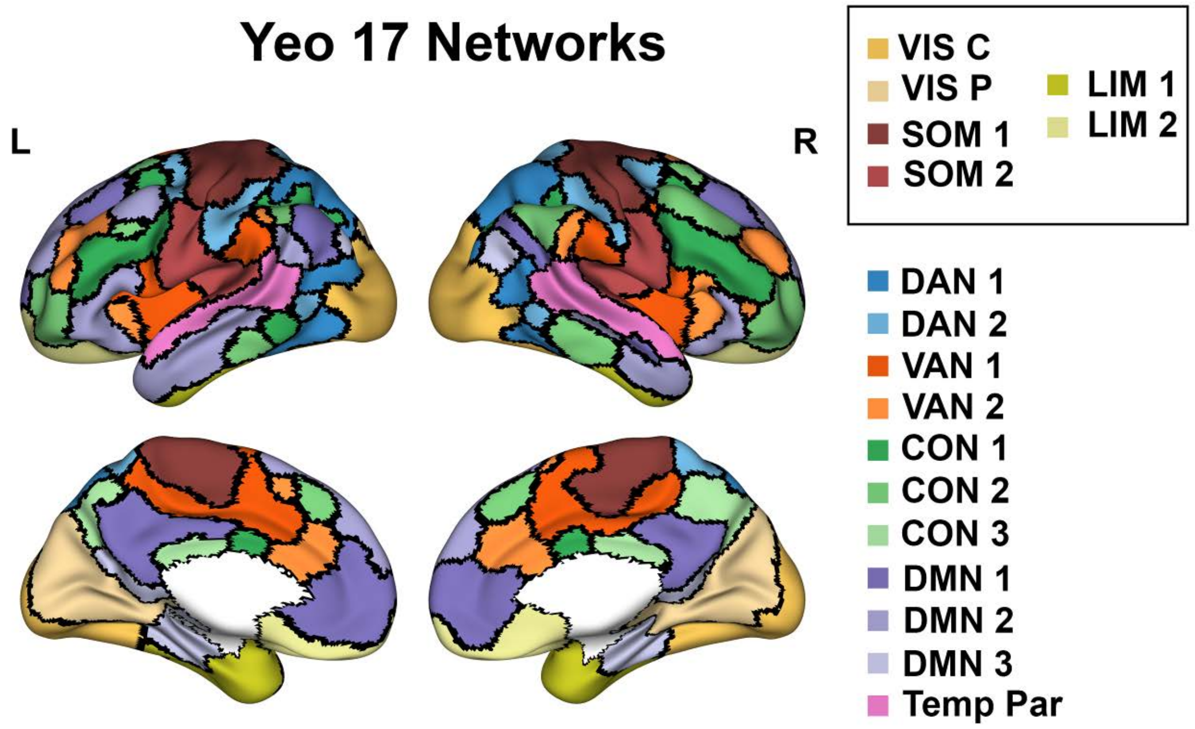

Supplementary Figure 1. *Schaefer 17 Yeo Networks*. Here we show the 17 Yeo networks across the Schaefer parcellation, defined by independent data. Following Chan et al. (2014), SyS measures in the main paper excluded the non-associative (sensory) regions shown upper box; results using all 17 networks are shown later in Supplementary Materials.

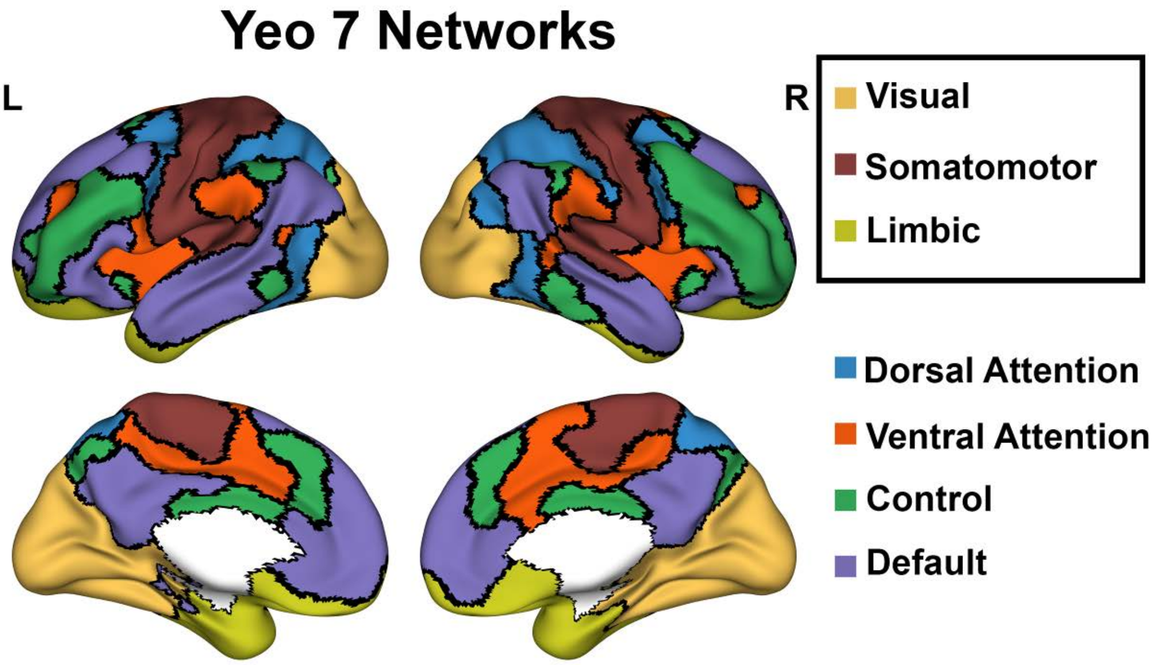

Supplementary Figure 2 *Schaefer 7 Yeo Networks*. Here we show the 7 Yeo networks across the Schaefer parcellation, defined by independent data. Following Chan et al. (2014), SyS measures in the main paper excluded the non-associative (sensory) regions shown upper box; results relating SyS to cognition using the 7 Yeo networks are shown below.

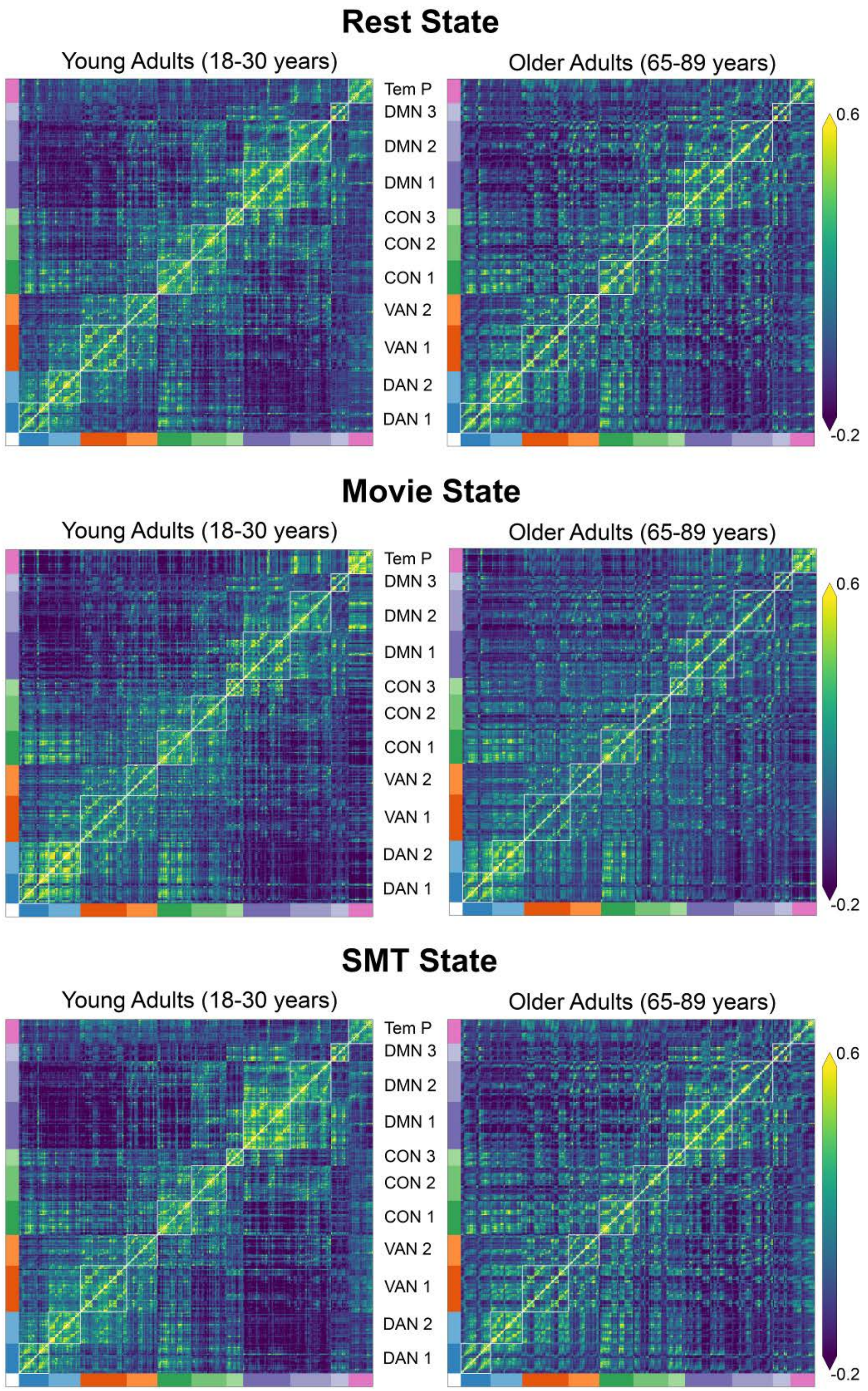

Supplementary Figure 3 *Average Connectivity Matrixes for three states (Rest, Movie and SMT) using 17 Yeo Networks***.** Plot shows average correlation matrices for a selection of young and older adults. Correlation coefficients were transformed to Z-scores before computing group averages and then inverted back to correlation coefficients for visualization purposes. White blocks indicate within network ROI connectivity values. Off-block values represent between networks connectivity. In Supplementary Figure 4, we show how within- and between-network connectivity vary with age.

Within, and Between network connectivity measures across age

We examined the association of within and between network connectivity and age for the three tasks. This was done to examine whether the association between SyS and age reported in the main paper was mainly driven by changes in within- and/or between-network connectivity (see Supplementary Fig. 4). We found that within-network connectivity decreased with age, mainly linearly, while between-network connectivity increased quadratically with age.

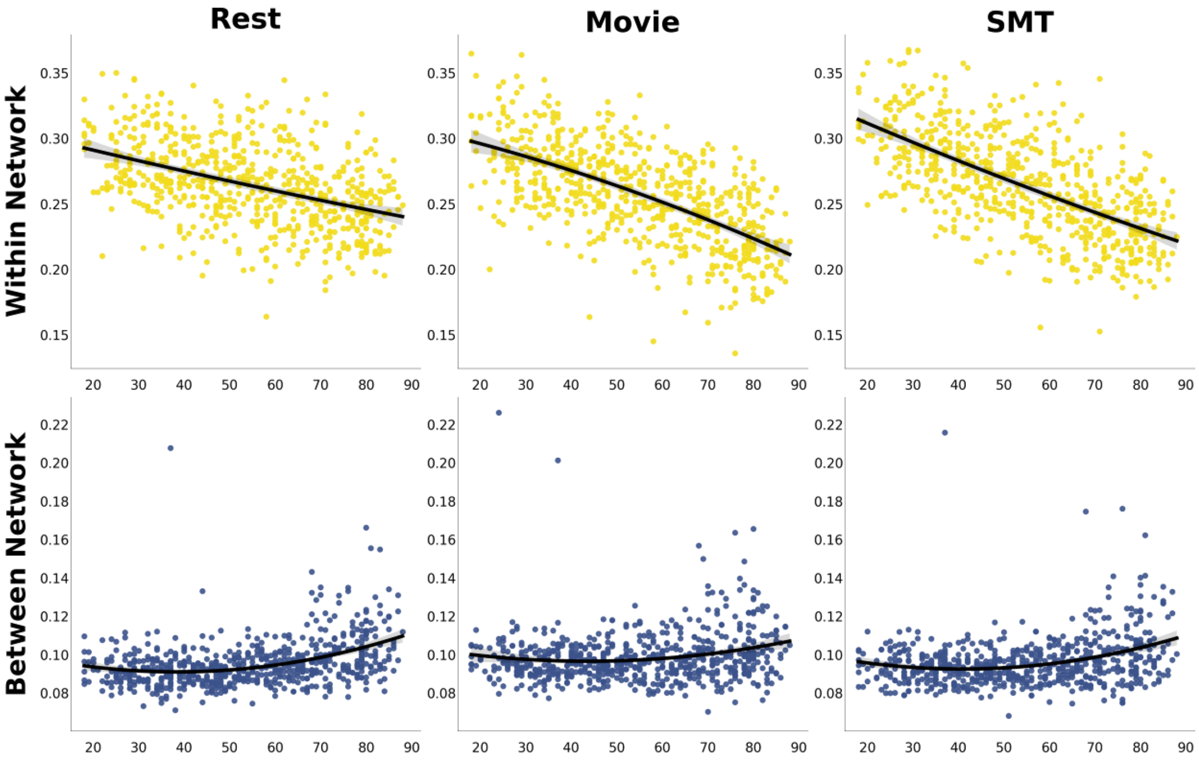

Supplementary Figure 4 *Connectivity across networks with GSR*. Plot shows relationship between within network, and between network connectivity and age. All averaging was performed on fisher-transformed correlations. Shaded area represents 95% confidence interval. The y-axis shows values on correlation scale.

Interaction Plots

We note that we observed a significant SyS*Sex*Age interaction when predicting Fluid Intelligence from SyS measured within the Rest task. We note that this interaction was not significant when controlling for Education and TIV (see below).

Fluid Intelligence

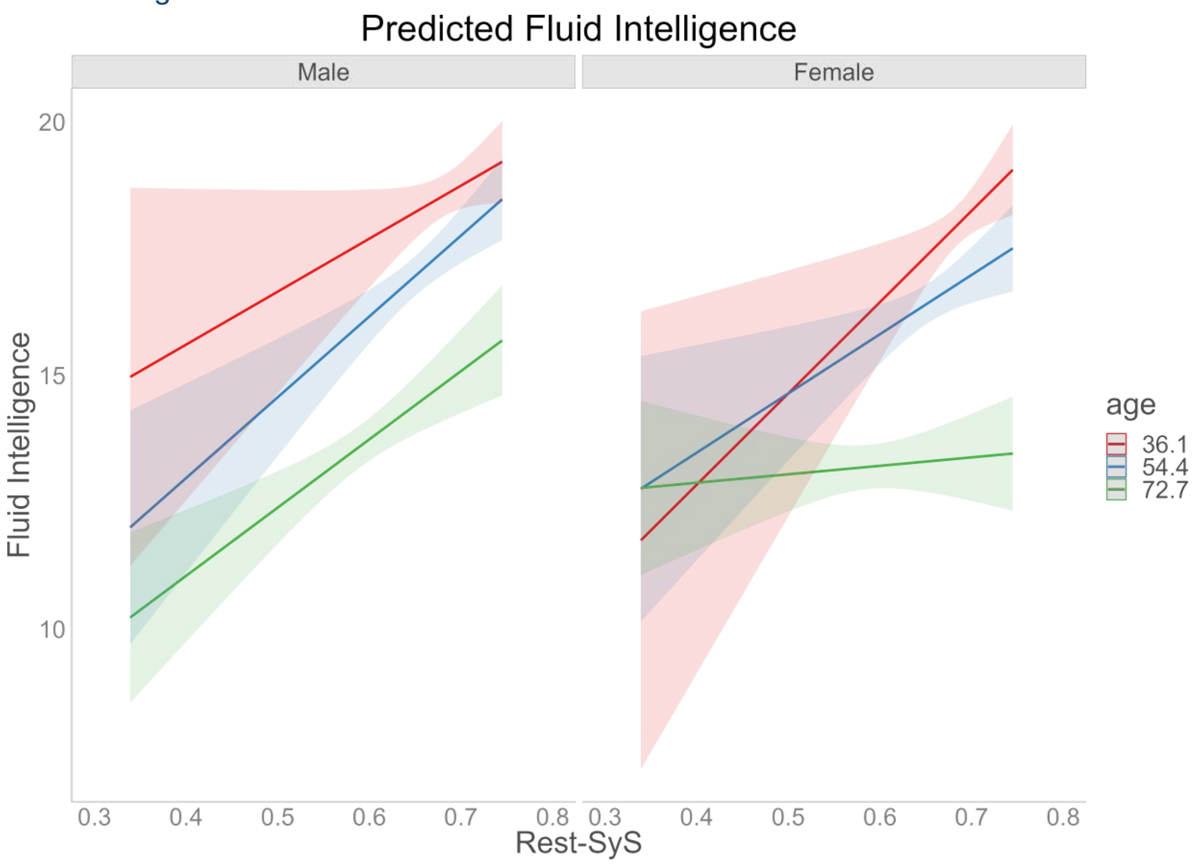

Supplementary Figure 5 *Illustration of SyS-by-Sex-by-Age interaction when predicting Fluid Intelligence from the Rest Task*. There was a significant SyS by Sex by linear Age interaction (β = -0.30 $t_{(615)}$= -2.05 p = 0.041; η^2 = 0.006)

Is SyS more strongly associated with Fluid Intelligence compared to Episodic Memory

To examine whether the relationship between SyS and cognition was different for our fluid intelligence and episodic memory measures, we ran additional model predicting SyS from age, sex and both fluid intelligence and memory. We found that SyS was more strongly associated with intelligence than memory in all three states [**Rest**: $\beta_{difference} = 0.16$, $t = 2.38$, $p = 0.018$; **Movie**: $\beta_{difference} = 0.21$, $t = 3.36$, $p < 0.001$; **SMT**: $\beta_{difference} = 0.18$, $t = 3.00$, $p = 0.002$].

Age and Cognition effects when SyS was computed using both associative and sensorimotor networks.

Here we computed SyS across all 17 Yeo networks. We show that including sensory motor networks in the SyS computation did not change the reported in the main report age and cognition effects.

Task	Polynomial Term				
	age^1	age^2	Sex	age^1:Sex	age^2:Sex
Rest ***R^2 = 0.43*** ***df = 621***	**ß = -0.03** **T = -21.21** **p < 0.001** **η^2 = 0.42**	**ß = -0.01** **T = -3.76** **p < 0.001** **η^2 = 0.02**	**ß = -0.00** **T = -2.61** **p = 0.009** **η^2 = 0.01**	ß = -0.00 T = -0.37 p = 0.71	ß = -0.00 T = -0.04 p = 0.97
Movie ***R^2 = 0.51*** ***df = 621***	**ß = -0.037** **T = -25.40** **p < 0.001** **η^2 = 0.51**	**ß = -0.01** **T = -4.60** **p < 0.001** **η^2 = 0.03**	ß = -0.00 T = -0.11 p = 0.91	ß = -0.00 T = -0.80 p = 0.42	ß = -0.000 T = -0.37 p = 0.73
SMT ***R^2 = 0.57*** ***df = 621***	**ß = -0.038** **T = -28.50** **p < 0.001** **η^2 = 0.57**	**ß = -0.004** **T = -3.68** **p < 0.001** **η^2 = 0.02**	ß = -0.002 T = -1.20 p = 0.23	ß = -0.00 T = -0.37 p = 0.71	ß = 0.001 T = 0.79 p = 0.43

Supplementary Table 1 *Predicting SyS from Age in full sample (N=627).* Statistics from linear regression models predicting SyS from sex, a second-order polynomial expansion of age and their interactions. SyS was computed including both associative and sensorimotor networks – all 17 Networks. The terms age^1 and age^2 refer to the linear and quadratic effects of age respectively. Significant effects are shown in **bold**.

Task	Terms					
	SyS	SyS:age[1]	SyS:age[2]	SyS:Sex	Sex:age[1]	Sex:age[2]
Rest<br>***R^2 = 0.49***<br>***df = 615***	**ß = 0.74**<br>**T = 5.28**<br>**p < 0.001**<br>**η² = 0.04**	ß = -0.20<br>T = -1.40<br>p = 0.16	ß = -0.10<br>T = -0.89<br>p = 0.37	ß = -0.10<br>T = -0.75<br>p = 0.45	ß = -0.00<br>T = -0.02<br>p = 0.98	ß = -0.10<br>T = -0.81<br>p = 0.41
Movie<br>***R^2 = 0.47***<br>***df = 615***	**ß = 0.47**<br>**T = 2.90**<br>**p = 0.003**<br>**η² = 0.02**	ß = -0.06<br>T = -0.39<br>p = 0.70	ß = -0.11<br>T = -0.94<br>p = 0.35	ß = -0.19<br>T = -1.20<br>p = 0.23	ß = -0.11<br>T = -0.64<br>p = 0.52	ß = 0.02<br>T = 0.16<br>p = 0.87
SMT<br>***R^2 = 0.48***<br>***df = 615***	**ß = 0.56**<br>**T = 3.21**<br>**p = 0.001**<br>**η² = 0.03**	ß = 0.014<br>T = 0.072<br>p = 0.94	ß = -0.22<br>T = -1.91<br>p = 0.05	ß = -0.15<br>T = -0.85<br>p = 0.39	ß = -0.07<br>T = -0.40<br>p = 0.69	ß = -0.12<br>T = -0.83<br>p = 0.41

Supplementary Table 2 *Predicting Fluid Intelligence from SyS in full sample (N=627).* Statistics from linear models predicting Fluid Intelligence from SyS, after adjusting for sex, second-order effects of age and their interaction. SyS was computed including both associative and sensorimotor networks.

Task	Terms					
	SyS	SyS:age[1]	SyS:age[2]	SyS:Sex	Sex:age[1]	Sex:age[2]
Rest<br>***R^2 = 0.16***<br>***df = 615***	**ß = 0.94**<br>**T = 3.14**<br>**p = 0.001**<br>**η² = 0.02**	ß = -0.19<br>T = -0.60<br>p = 0.55	ß = -0.16<br>T = -0.70<br>p = 0.48	ß = 0.08<br>T = 0.27<br>p = 0.79	ß = -0.09<br>T = -0.29<br>p = 0.78	ß = 0.26<br>T = 0.96<br>p = 0.34
Movie<br>***R^2 = 0.16***<br>***df = 615***	ß = 0.36<br>T = 1.06<br>p = 0.29<br>η² = 0.0002	ß = -0.55<br>T = -1.55<br>p = 0.12	ß = -0.14<br>T = -0.58<br>p = 0.56	ß = -0.48<br>T = -1.42<br>p = 0.16	ß = -0.63<br>T = -1.76<br>p = 0.08	ß = 0.32<br>T = 1.06<br>p = 0.29
SMT<br>***R^2 = 0.16***<br>***df = 615***	ß = 0.68<br>T = 1.86<br>p = 0.06<br>η² = 0.005	ß = -0.36<br>T = -0.91<br>p = 0.37	ß = -0.14<br>T = -0.58<br>p = 0.56	ß = -0.21<br>T = -0.58<br>p = 0.56	ß = -0.45<br>T = -1.14<br>p = 0.25	ß = 0.34<br>T = 1.05<br>p = 0.30

Supplementary Table 3 *Predicting Episodic Memory from SyS in the full sample (N=627).* Statistics from linear models predicting Episodic Memory from SyS, after adjusting for sex, second-order effects of age and their interaction. SyS was computed including both associative and sensorimotor networks.

Age and Cognition effects when we control for FD at the group level

Here we show that the age and cognition effects reported in the main manuscript remain when controlling for participant average FD displacement. Although the association between SyS measures and age and cognition showed lower t-statistics, it still remained significant.

Task	Polynomial Term				
	age¹	age²	Sex	age¹:Sex	age²:Sex
Rest ***R^2 = 0.53*** ***df = 620***	**ß = -0.024** **T = -12.80** **p < 0.001** **η^2 = 0.21**	**ß = -0.009** **T = -5.49** **p < 0.001** **η^2 = 0.05**	**ß = 0.003** **T = 1.76** **p = 0.078** **η^2 = 0.006**	ß = 0.001 T = 0.37 p = 0.71	ß = 0.001 T = 0.55 p = 0.58
Movie ***R^2 = 0.67*** ***df = 620***	**ß = -0.029** **T = -16.26** **p < 0.001** **η^2 = 0.30**	**ß = -0.01** **T = -3.83** **p < 0.001** **η^2 = 0.02**	ß = 0.003 T = 1.89 p = 0.05	ß = -0.001 T = -0.35 p = 0.73	ß = -0.003 T = -1.66 p = 0.09
SMT ***R^2 = 0.63*** ***df = 620***	**ß = -0.035** **T = -18.38** **p < 0.001** **η^2 = 0.35**	**ß = -0.009** **T = -5.61** **p < 0.001** **η^2 = 0.05**	ß = 0.002 T = 1.33 p = 0.18	ß = 0.001 T = 0.58 p = 0.57	ß = 0.001 T = 1.08 p = 0.28

Supplementary Table 4 *Predicting SyS from Age in full sample (N=627) controlling for FD.* Statistics from linear regression models predicting SyS from FD, sex, a second-order polynomial expansion of age and their interactions. The terms age¹ and age² refer to the linear and quadratic effects of age respectively. Significant effects are shown in **bold**.

Task	Terms					
	SyS	SyS:age¹	SyS:age²	SyS:Sex	Sex:age¹	Sex:age²
Rest ***R^2 = 0.49*** ***df = 614***	**ß = 0.69** **T = 4.48** **p < 0.001** **η^2 = 0.028**	ß = -0.21 T = -1.40 p = 0.16	ß = -0.10 T = -0.88 p = 0.38	ß = -0.09 T = -0.66 p = 0.50	ß = -0.01 T = -0.09 p = 0.93	ß = -0.14 T = -1.13 p = 0.26
Movie ***R^2 = 0.48*** ***df = 614***	**ß = 0.52** **T = 2.82** **p = 0.004** **η^2 = 0.012**	ß = -0.15 T = -0.93 p = 0.34	ß = -0.10 T = -0.84 p = 0.40	ß = -0.19 T = -1.25 p = 0.21	ß = -0.11 T = -0.69 p = 0.49	ß = -0.01 T = -0.11 p = 0.91
SMT ***R^2 = 0.48*** ***df = 614***	**ß = 0.54** **T = 2.87** **p = 0.004** **η^2 = 0.021**	ß = 0.07 T = 0.37 p = 0.71	ß = -0.23 T = -1.88 p = 0.06	ß = -0.25 T = -1.44 p = 0.15	ß = -0.15 T = -0.85 p = 0.40	ß = -0.10 T = -0.72 p = 0.47

Supplementary Table 5 *Predicting Fluid Intelligence from SyS in full sample (N=627) controlling for FD.* Statistics from linear models predicting Fluid Intelligence from SyS, after adjusting for FD, sex, second-order effects of age and their interaction.

Task	Terms					
	SyS	SyS:age[1]	SyS:age[2]	SyS:Sex	Sex:age[1]	Sex:age[2]
Rest<br>***R^2 = 0.16***<br>***df = 614***	**ß = 0.74**<br>**T = 2.28**<br>**p = 0.023**<br>**η^2 = 0.007**	ß = -0.27<br>T = -0.85<br>p = 0.39	ß = -0.15<br>T = -0.60<br>p = 0.55	ß = -0.10<br>T = -0.33<br>p = 0.74	ß = -0.20<br>T = -0.67<br>p = 0.50	ß = 0.27<br>T = 1.01<br>p = 0.31
Movie<br>***R^2 = 0.16***<br>***df = 614***	ß = 0.22<br>T = 0.58<br>p = 0.56<br>η^2 = 0.0001	ß = -0.39<br>T = -1.13<br>p = 0.26	ß = -0.16<br>T = -0.66<br>p = 0.51	ß = -0.43<br>T = -1.31<br>p = 0.19	ß = -0.55<br>T = -1.64<br>p = 0.10	ß = 0.35<br>T = 1.27<br>p = 0.20
SMT<br>***R^2 = 0.16***<br>***df = 614***	ß = 0.31<br>T = 1.09<br>p = 0.28<br>η^2 = 0.002	ß = -0.30<br>T = -0.78<br>p = 0.44	ß = -0.20<br>T = -0.78<br>p = 0.44	ß = -0.42<br>T = -1.18<br>p = 0.24	ß = -0.51<br>T = -1.39<br>p = 0.17	ß = 0.35<br>T = 1.15<br>p = 0.25

Supplementary Table 6 *Predicting Episodic Memory from SyS in the full sample (N=627) controlling for FD.* Statistics from linear models predicting Episodic Memory from SyS, after adjusting for FD, sex, second-order effects of age and their interaction.

Controlling for Education and TIV

Based on a reviewer suggestion, we ran additional models in the full sample to examine whether the association between SyS and cognition would remain significant if we control for total intracranial volume (TIV) and education. For education, we had a four-level variable that indicated whether participants had no education (0), or had completed (1) GSCE/O-level, (2) A- level or (3) University level education. Both TIV and Education were positively associated with fluid intelligence. Education, but not TIV, was positively associated with Episodic Memory. Most importantly, even after controlling for Education and TIV, all three measures of SyS remained related to Fluid intelligence. Similarly, the association between Rest-SyS and episodic memory remained significant after adding Education and TIV in the model.

Task	Terms					
	SyS	SyS:age[1]	SyS:age[2]	SyS:Sex	TIV	Education
Rest<br>***R^2 = 0.56***<br>***df = 612***	**ß = 0.60**<br>**T = 4.70**<br>**p < 0.001**<br>**η^2 = 0.03**	ß = -0.23<br>T = -1.63<br>p = 0.10	ß = -0.001<br>T = -0.08<br>p = 0.94	ß = -0.04<br>T = -0.28<br>p = 0.78	**ß = 0.35**<br>**T = 2.81**<br>**p = 0.005**<br>**η^2 = 0.01**	**ß = 0.84**<br>**T = 8.46**<br>**p < 0.001**<br>**η^2 = 0.1**
Movie<br>***R^2 = 0.55***<br>***df = 612***	**ß = 0.51**<br>**T = 3.56**<br>**p = 0.001**<br>**η^2 = 0.02**	ß = -0.13<br>T = -0.90<br>p = 0.37	ß = 0.001<br>T = 0.01<br>p = 0.99	ß = -0.17<br>T = -1.17<br>p = 0.24	**ß = 0.38**<br>**T = 3.068**<br>**p = 0.002**<br>**η^2 = 0.02**	**ß = 0.86**<br>**T = 8.69**<br>**p < 0.001**<br>**η^2 = 0.1**
SMT<br>***R^2 = 0.55***<br>***df = 612***	**ß = 0.47**<br>**T = 2.98**<br>**p = 0.003**<br>**η^2 = 0.02**	ß = -0.01<br>T = -0.05<br>p = 0.95	ß = -0.11<br>T = -0.96<br>p = 0.33	ß = -0.16<br>T = -1.07<br>p = 0.29	**ß = 0.38**<br>**T = 3.06**<br>**p = 0.002**<br>**η^2 = 0.02**	**ß = 0.83**<br>**T = 8.24**<br>**p < 0.001**<br>**η^2 = 0.1**

Supplementary Table 7 *Predicting Fluid Intelligence from SyS in full sample (N=627) controlling for TIV and Education.* Statistics from linear models predicting Fluid Intelligence from SyS, after adjusting for TIV, education, sex, second-order effects of age and their interaction.

Task	Terms					
	SyS	SyS:age[1]	SyS:age[2]	SyS:Sex	TIV	Education
Rest ***R^2 = 0.20*** ***df = 612***	**ß = 0.70** **T = 2.44** **p = 0.01** **η^2 = 0.008**	ß = -0.29 T = -0.94 p = 0.34	ß = -0.05 T = -0.19 p = 0.85	ß = -0.01 T = -0.045 p = 0.96	ß = 0.22 T = 0.80 p = 0.43	**ß = 1.13** **T = 5.10** **p < 0.001** **η^2 = 0.004**
Movie ***R^2 = 0.20*** ***df = 612***	ß = 0.16 T = 0.49 p = 0.62 η^2 = 0.0000	ß = -0.37 T = -1.11 p = 0.27	ß = -0.04 T = -0.16 p = 0.87	ß = -0.39 T = -1.22 p = 0.22	ß = 0.25 T = 0.91 p = 0.36	**ß = 1.17** **T = 5.32** **p < 0.001**
SMT ***R^2 = 0.20*** ***df = 612***	ß = 0.32 T = 0.92 p = 0.36 η^2 = 0.001	ß = -0.41 T = -1.10 p = 0.27	ß = -0.05 T = -0.21 p = 0.84	ß = -0.31 T = -0.90 p = 0.37	ß = 0.23 T = 0.82 p = 0.41	**ß = 1.17** **T = 5.26** **p < 0.001**

Supplementary Table 8 *Predicting Episodic Memory from SyS in the full sample (N=627) controlling for TIV and Education.* Statistics from linear models predicting Episodic Memory from SyS, after adjusting for TIV, education, sex, second-order effects of age and their interaction.

Effects of Number of ROIs and Networks

Here we examined how the relationships between SyS measures and age, and between SyS measures and cognition, were affected by using different resolution of the Schaefer Parcellation, or by using, the 7 Yeo networks rather than the 17 Yeo networks. In Supplementary Figure 6, we plot the combined (linear and quadratic) age effect on SyS measured with different number ROIs and Networks. There was not an obvious systematic effect of number of ROIs, though the age effects (measured with partial eta squared) were generally stronger for 17 than 7 networks, at least for SMT and Rest. Nonetheless, effects of age were consistently large in all cases.

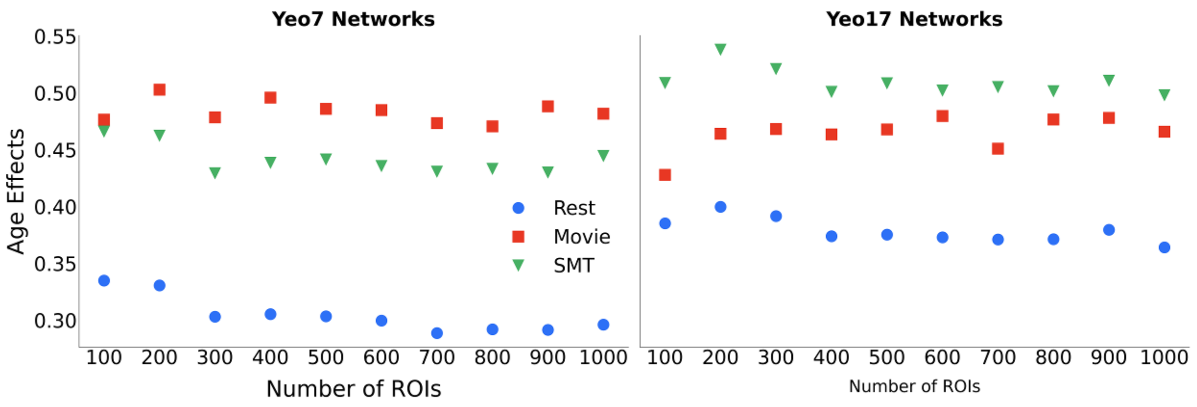

Supplementary Figure 6 *Figure shows how effect size – partial eta squared η^2 of combined linear and quadratic age on different task SyS measures differs across different number of ROIs used to compute the functional connectivity.* On the left, we show the age effects on SyS measures computed from the 100-1000 ROIs using the Schaefer Parcellation with ROI being grouped into the 7 Yeo networks. On the right, we show the age effect on SyS measures computed from the 17 Yeo Networks.

In Supplementary Figures 7 and 8, we show the effect SyS has on fluid intelligence and episodic memory when controlling for age and sex effects. This time, there was a trend for higher effect sizes as the number of ROIs increased, as well as when the number of networks increased (with the exception of the Movie state, which did not seem to increase with more networks, similar to the effect of age above). Nonetheless, although small, the effect size for fluid intelligence was significant in all cases.

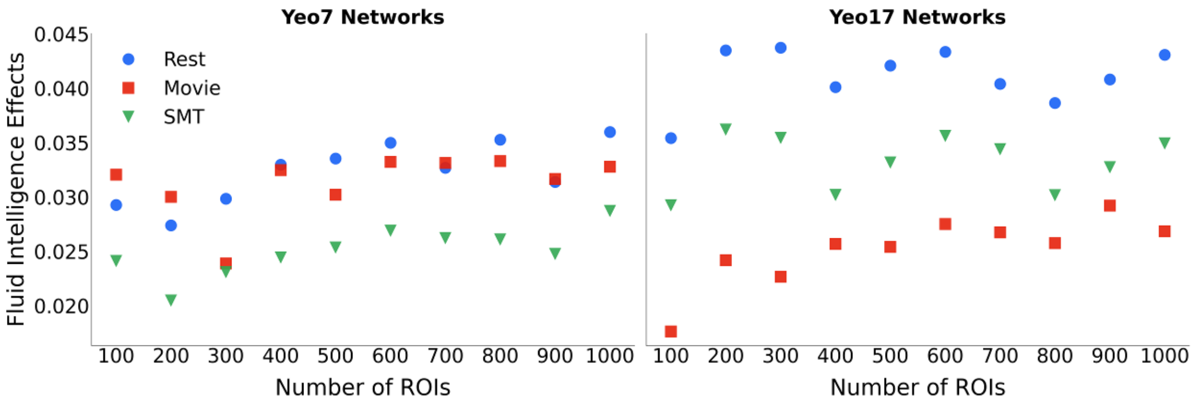

Supplementary Figure 7 *Figure shows how the partial eta squared of the relationship between SyS and Fluid Intelligence differs across different ROIs and Networks.*

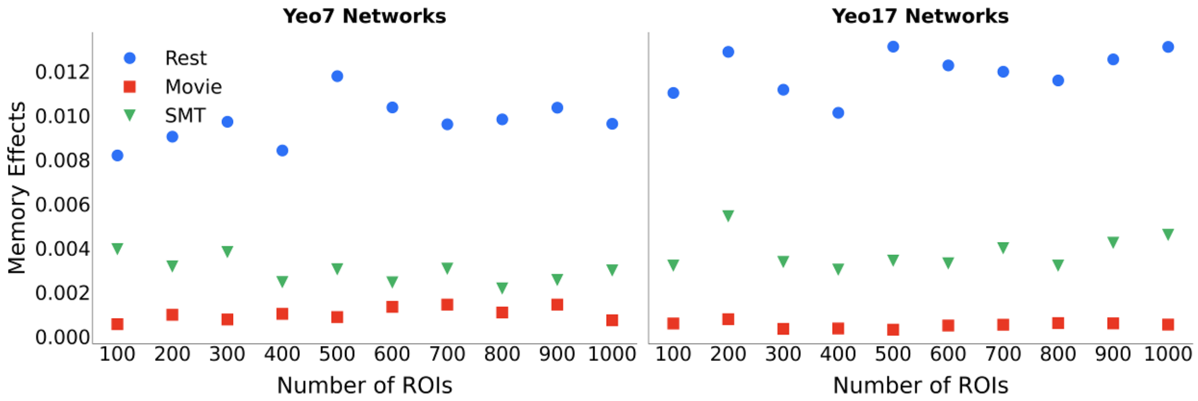

Supplementary Figure 8. *Figure shows how the partial eta squared of the relationship between SyS and Episodic Memory differs across different number of ROIs and Networks.*

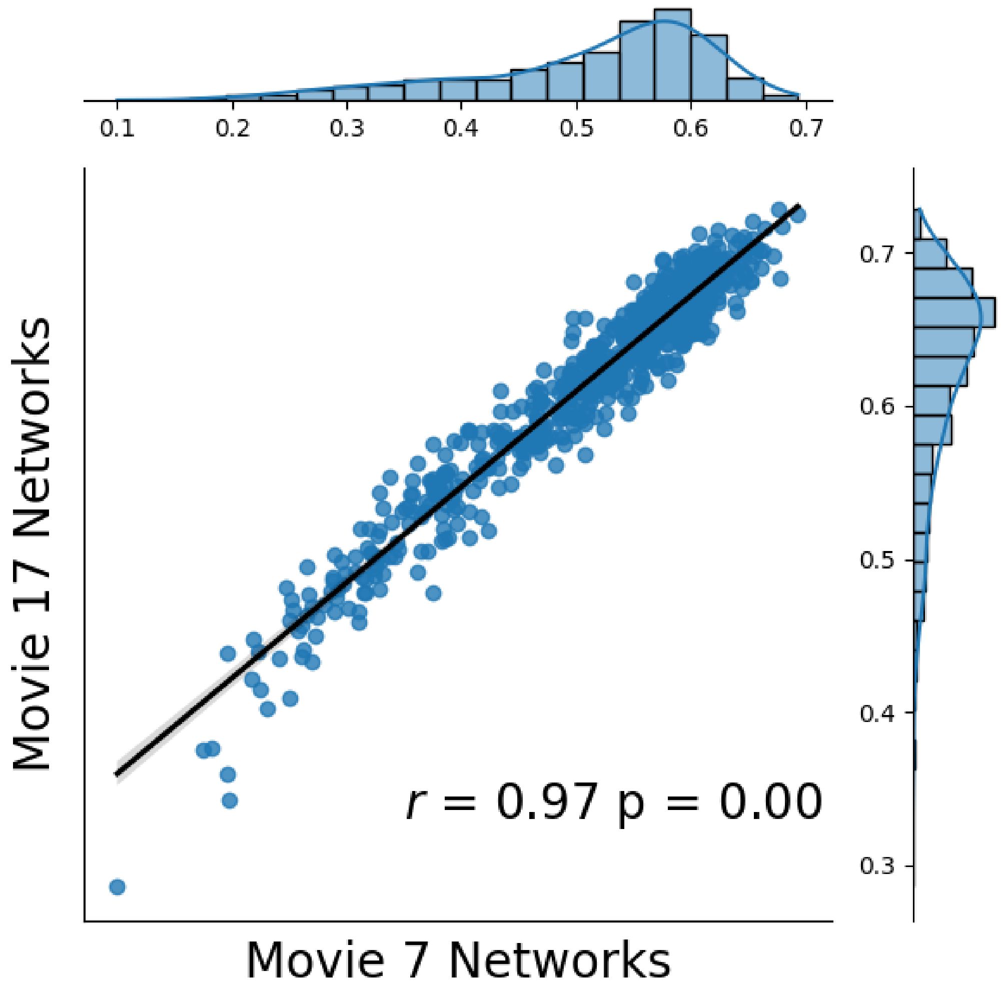

Supplementary Figure 9 *Figure shows correlation between SyS values computed with 500 ROIs with 7 and 17 Yeo Networks for the Movie task*. Each dot is the SyS value for a single subject. Y axis and right histogram shows SyS values computed with 17 Yeo networks. X axis shows SyS values computed with 7 Yeo networks.

Association between SyS, Age and Cognition using 7 Yeo Networks and 500 ROIs

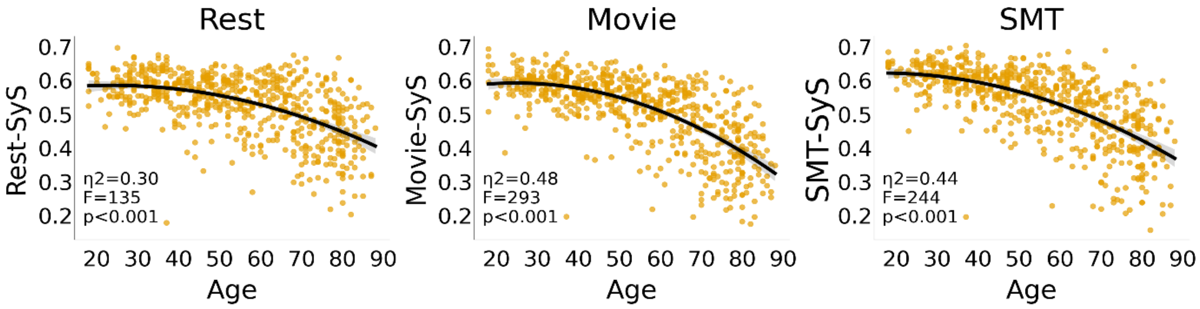

Supplementary Figure 10 *Predicting SyS from Age in full sample (N=627) using 7 Yeo Networks.* SyS decreases with age in each of the three brain states. Black line indicates second-order fit, and grey area demonstrates 95% bootstrapped intervals for the fit. Robust regression showed the age effects remained significant when accounting for possible "outlier" values.

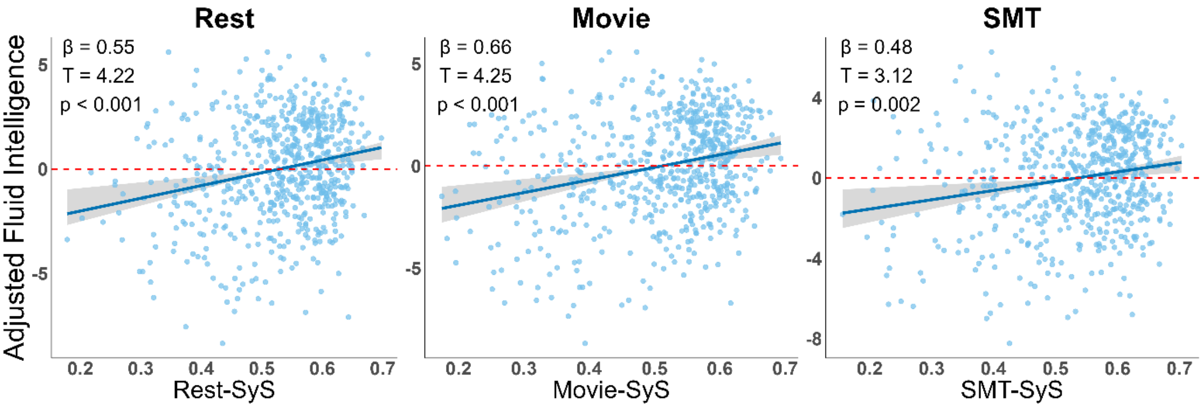

Supplementary Figure 11 *Predicting Fluid Intelligence from SyS in full sample (N=627) using 7 Yeo networks.* Fluid intelligence was positively related to SyS in each of the three brain states, after adjusting for second-order effects of age (and sex, and interactions between all three variables).

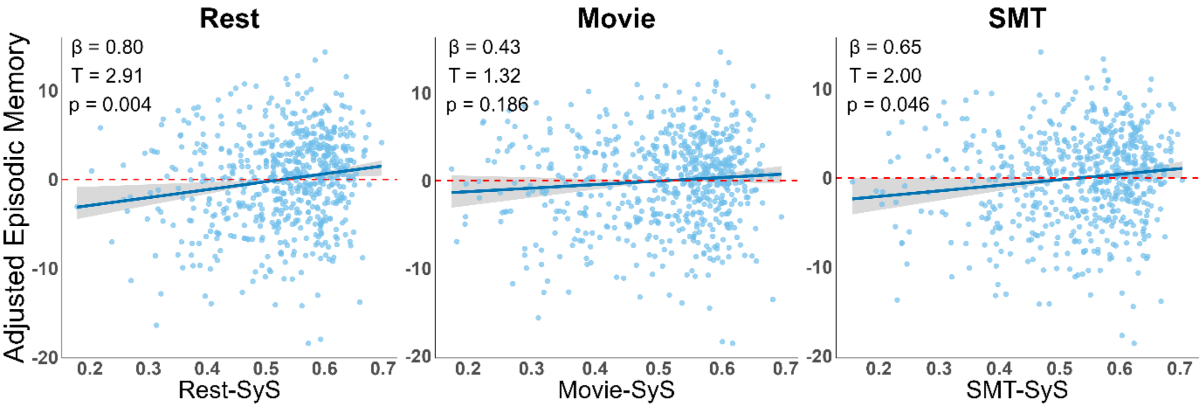

Supplementary Figure 12 *Predicting Episodic Memory from SyS in full sample (N=627) using 7 Yeo Networks.* Episodic memory was significantly related to SyS in the Rest and SMT states.

Association between SyS, MA and late-life cognition with 7 Yeo Networks

Here we show how MA was associated with SyS when we used 7 Yeo networks rather than 17 networks. Supplementary Table 9 shows the regressions of SyS for each state (now using 7 networks) on MA, age and sex; only SyS in the SMT state showed an effect of MA.

Task	Terms		
	MA	age[1]	Sex
Rest<br>***R^2 = 0.09***<br>***df = 188***	ß = 0.01<br>T = 1.60<br>p = 0.11	**ß = -0.03**<br>**T = -4.17**<br>**p < 0.001**<br>**η^2 = 0.059**	ß = -0.00<br>T = -0.18<br>p = 0.86
Movie<br>***R^2 = 0.15***<br>***df = 188***	ß = 0.01<br>T = 1.72<br>p = 0.09	**ß = -0.04**<br>**T = -5.74**<br>**p < 0.001**<br>**η^2 = 0.155**	ß = -0.00<br>T = -0.29<br>p = 0.877
SMT<br>***R^2 = 0.10***<br>***df = 188***	**ß = 0.015**<br>**T = 2.01**<br>**p = 0.045**	**ß = -0.03**<br>**T = -4.30**<br>**p < 0.001**<br>**η^2 = 0.078**	ß = -0.00<br>T = -0.28<br>p = 0.78

Supplementary Table 9 *Predicting SyS from MA in older adults (N=192)*. Statistics from linear models predicting SyS from MA, after adjusting for second-order effects of age and sex. See Table 1 legend for more details.

Furthermore, similar to the results reported in the manuscript, structural equation modelling did not reveal evidence that SyS, computed with 7 Yeo networks, mediated the effect of MA on late life cognition in any state (see Supplementary Fig. 13). The percent of variance mediated, calculated as indirect effect / total effect was 4.2%, Z = 1.37, p = 0.17 for Rest; 4.3%, Z = 1.40, p = 0.16 for Movie and 4.6%, Z = 1.47, p = 0.14 for SMT.

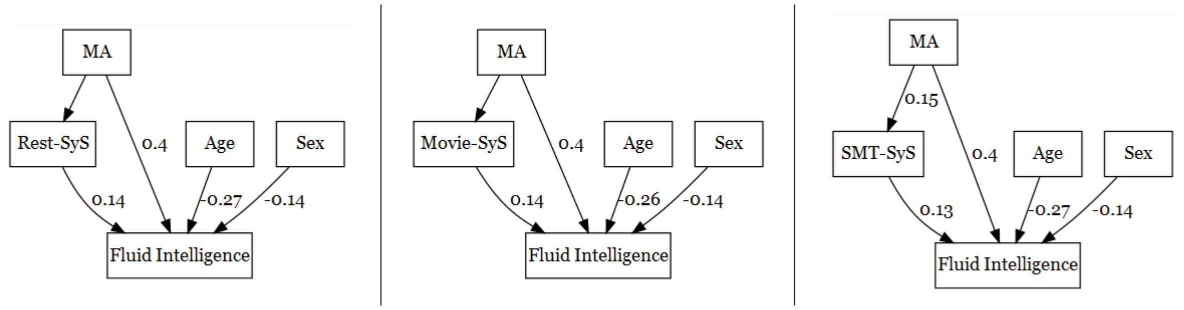

Supplementary Figure 13 *SyS from any state does not mediate the relationship between MA and Cattell in older adults (N=192), when using 7 Yeo Networks*. Path diagram showing standardised coefficients that were significant in the mediation analysis in older adults using SyS, for each of the three states.

Exploratory analyses with 17 Yeo Networks

As requested by a reviewer, we also report how MA and YS relates to age. The MA activities measure was not associated with age either when we looked at the whole sample (ß = -0.06; t = -0.09, p = 0.93; η^2 = 0.00), nor when we focused on the older adults (ß = -0.25; t = -0.58, p = 0.57; η^2 = 0.00). However, we did observe a significant negative association between YS and age in the full sample (ß = -0.85; t = -6.44, p < 0.001; η^2 = 0.07) that did not reach significance when focusing on the older adults (ß = -0.40; t = -1.66, p = 0.099; η2 = 0.014). We additionally, examined the association between late-life SyS and MA without regressing out age; again we only observed a significant association between SMT-SyS and MA [Rest: ß = 0.01, t = 1.72, p = 0.08; Movie: ß = 0.01, t = 1.85, p = 0.06; **SMT**: ß = 0.01; t = 2.26; p = 0.02].

We also show the results from exploratory analyses where we examined how early-life activities (education) relate to late-life SyS.

We found that youth-specific (YS) life activities were not consistently related across states to late-life system segregation. Using the 17 Yeo networks, we only found a significant association between YS and SyS that was above age in the SMT state: Rest (ß = 0.007, $t_{(188)}$ = 1.69, p = 0.09), Movie (ß = 0.006, $t_{(188)}$ = 1.24, p = 0.22) and **SMT** (ß = 0.010, $t_{(188)}$ = 2.18, p = 0.031). When we did not regress out age and sex, this YA-SyS relationship also reached significance for the Rest state: **Rest** (ß = 0.009, $t_{(188)}$ = 2.09, p = 0.03), Movie (ß = 0.008, $t_{(188)}$ = 1.71, p = 0.09) and **SMT** (ß = 0.012, $t_{(188)}$ = 2.56, p = 0.011). However, we note that these latter results are difficult to interpret as the significant association may be partly driven by the shared variance between YS and age.

Furthermore, although we found association between YS and SyS in SMT and Rest states, we still did not find a significant mediation effect (see Figure Below). The percent of variance mediated was 5.3%, Z = 1.53, p = 0.13 for Rest; 3.9%, Z = 1.33, p = 0.18 for Movie and 6.5%, Z = 1.71, p = 0.08 for SMT.

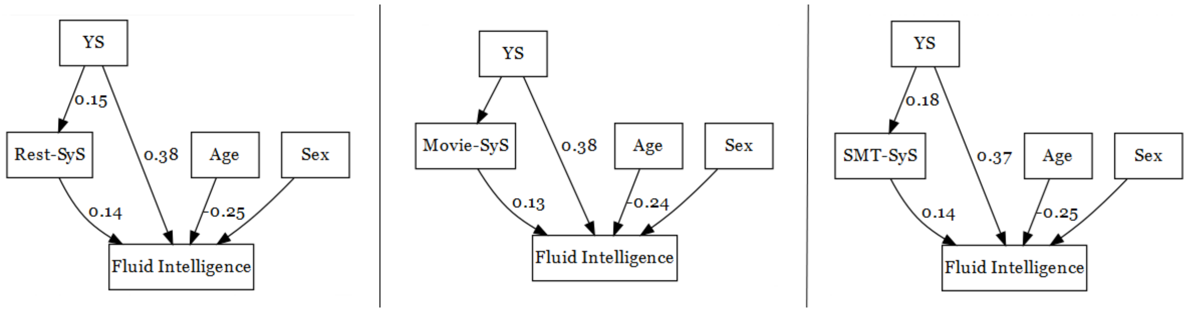

Supplementary Figure 14. *SyS from any state does not mediate the relationship between YS and Cattell in older adults (N=192), when using 17 Yeo Networks*. Path diagram showing standardised coefficients that were significant in the mediation analysis in older adults using SyS, for each of the three states.

Different Atlases

We additionally computed SyS using different brain atlases to test whether the effects reported in the main paper are specific to certain ROI definitions. More specifically, we compared the above results with the Schaefer ROIs to those using the Craddock atlas (Craddock et al., 2012) and the Gordon atlas (Gordon et al., 2016). Across both atlases, we found that SyS declines with age and is associated with Fluid intelligence, even after accounting for age and sex effects. For neither of the atlases did we find an association between SyS and midlife activities in older adults.

Craddock Atlas

Using the Craddock atlas (Craddock et al., 2012), we extracted data from 832 ROIs. Unlike the Yeo networks (defined over Schaefer ROIs), which were defined from independent data, here we defined the networks (over Craddock ROIs) in a data-driven fashion, i.e, from the same Cam-CAN data used in the main paper, using a consensus partitioning algorithm (Lancichinetti & Fortunato, 2012) as described in prior work on the Cam-CAN dataset (Geerligs et al., 2015). This might have the benefit that the ROIs are more age-representative, since many other network definitions are defined on young groups of participants. As in the main report, we focused on ROIs that belonged to 'associative' networks, namely the Dorsal Attention Network (DAN), Frontal Executive Network (FEN), anterior insula network (aInsula), Default mode network (DMN), Cingulate, Fronto-Parietal Control Network (FPCN), Precuneus (Prec), and ventral attention network (VAN). The networks are plotted in Supplementary Figure 15 and the correlation matrices are plotted in Supplementary Figure 16.

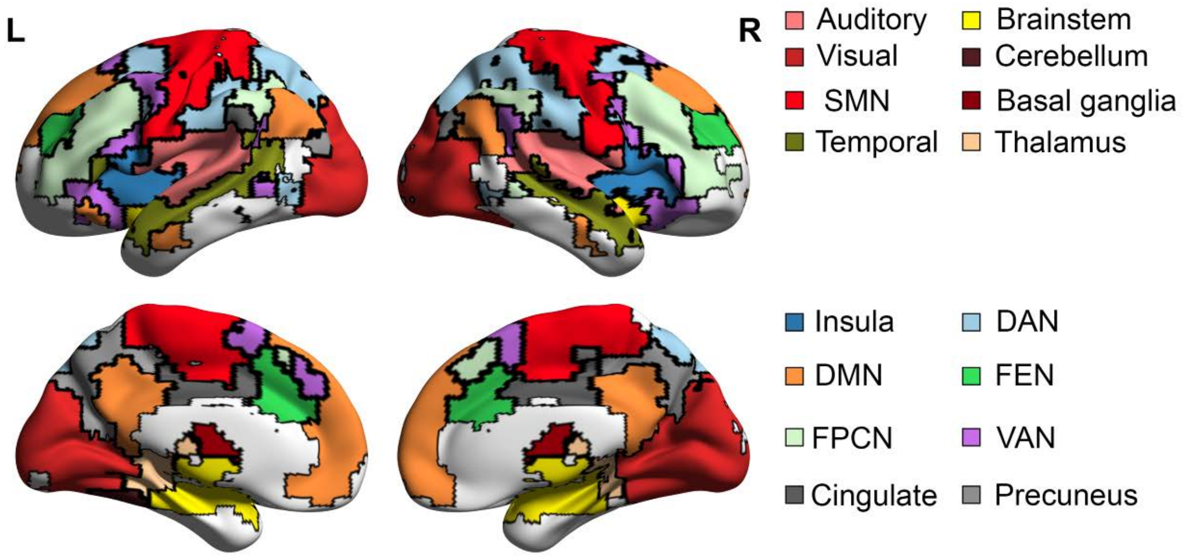

Supplementary Figure 15. *Craddock Networks*. The plot shows the age-representative networks created in Geerligs et al., (2015) from the same data, starting with the Craddock brain parcellation. Following Chan et al. (2014), SyS measures excluded the non-associative networks (shown at top of legend). We focused our analyses on associative networks: Insula, DAN, DMN, FEN, FPCN, VAN, Cingulate and Precuneus (shown at bottom of legend).

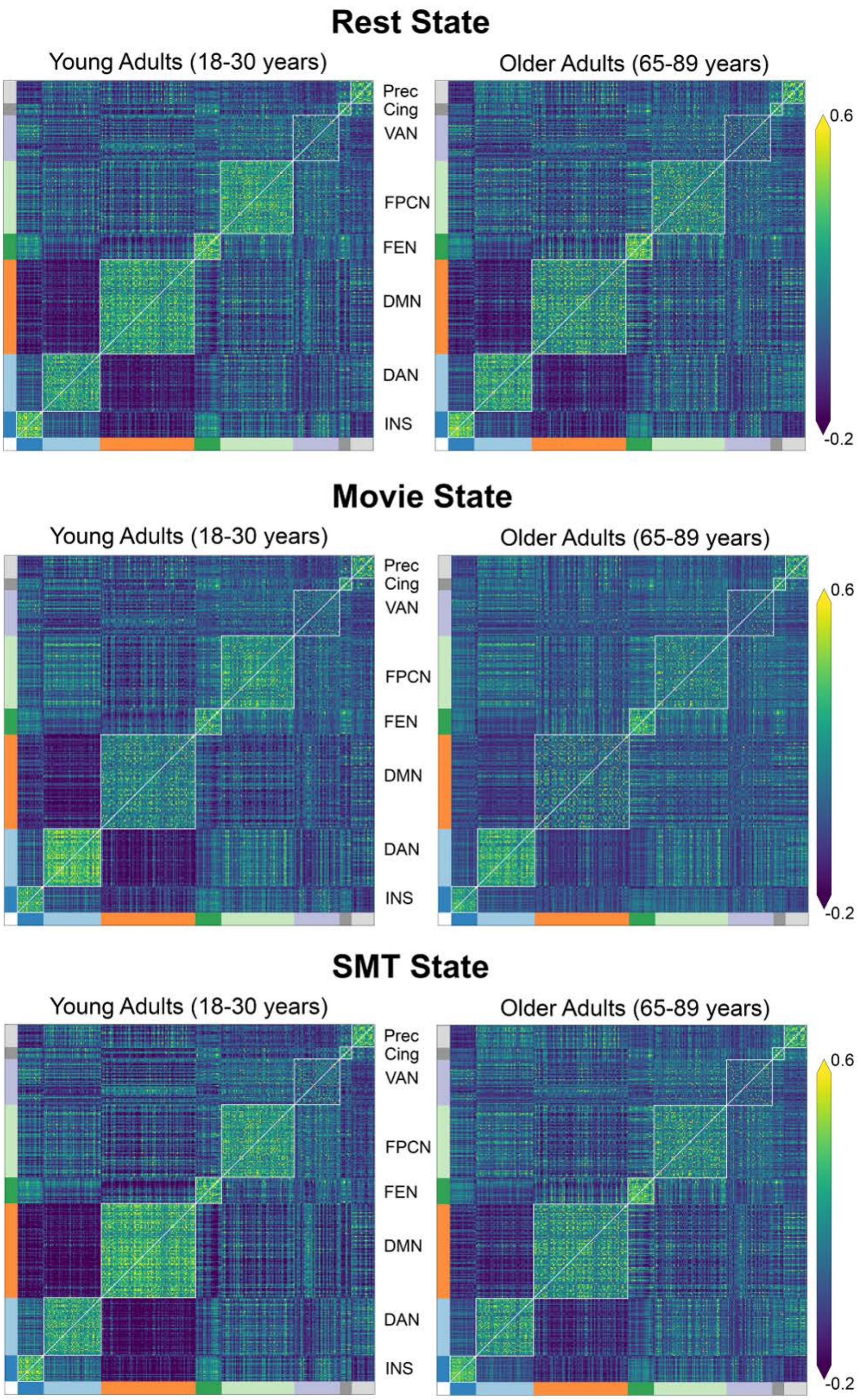

Supplementary Figure 16 *Average Connectivity Matrices using Craddock ROIs and data-driven network definition for the three states (Rest, Movie and SMT)*. Plot shows average correlation matrices for a selection of young and older adults. Correlation coefficients were transformed to Z-scores before computing group averages and then inverted back to correlation coefficients for visualization purposes. White blocks indicate within network ROI connectivity values. Off-block values represent between networks connectivity. Note tendency for higher off-block values and smaller within-block values in older adult group compared to the younger adults.

Age and Cognition Effects (Craddock Atlas)

Figures and Tables for the Craddock atlas corresponding to those in the main paper (using Schaefer atlas) are shown below. The main results are replicated, particularly for fluid intelligence. We also observe a significant association between Episodic Memory and Rest-SyS.

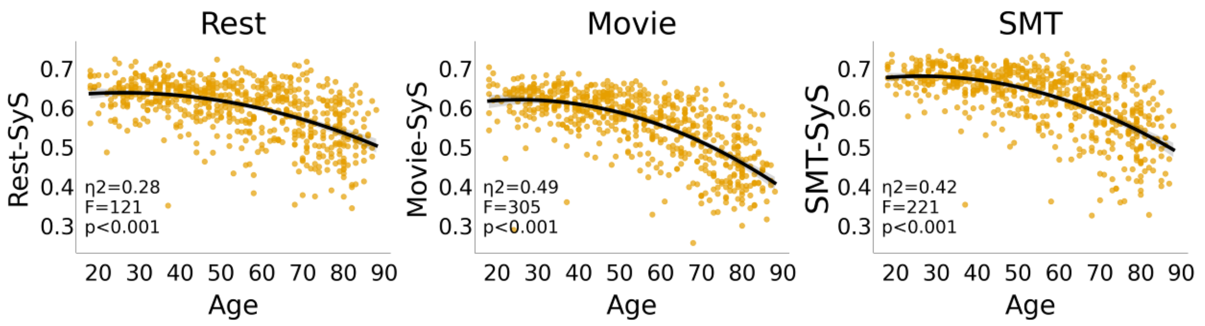

Supplementary Figure 17 *Predicting SyS from Age in full sample (N=627) using Craddock Parcellation.* SyS decreases with age in each of the three brain states.

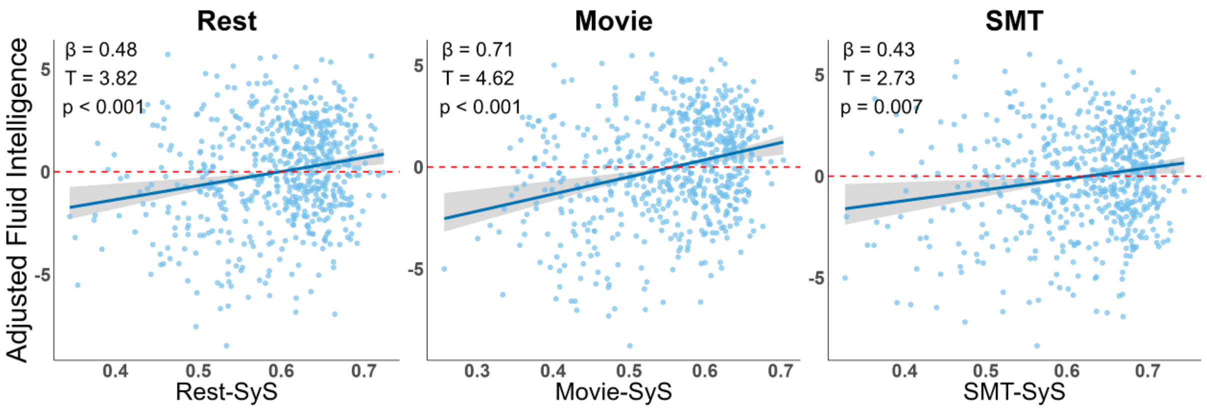

Supplementary Figure 18 *Predicting Fluid Intelligence from SyS in full sample (N=627) using Craddock Parcellation.* Fluid intelligence was positively related to SyS in each of the three brain states.

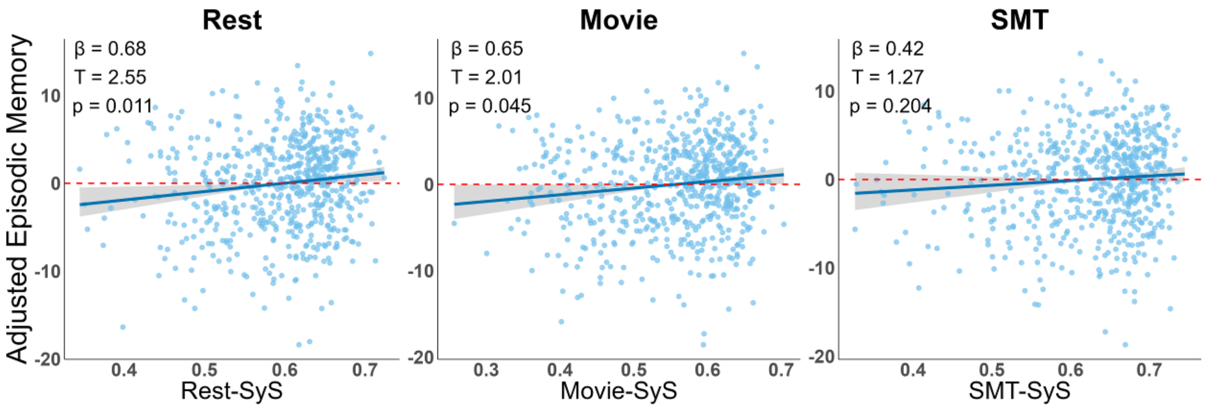

Supplementary Figure 19. *Predicting Episodic Memory from SyS in full sample (N=627) using Craddock Parcellation.* Rest-SyS was related to episodic memory, but no positive effect was observed for SyS from the other brain states.

Task	Polynomial Term				
	age^1	age^2	Sex	age^1:Sex	age^2:Sex
Rest<br>***R^2 = 0.28***<br>***df = 621***	**ß = -0.04**<br>**T = -14.95**<br>**p < 0.001**<br>**η^2 = 0.26**	**ß = -0.01**<br>**T = -4.38**<br>**p < 0.001**<br>**η^2 = 0.03**	ß = 0.003<br>T = 1.36<br>p = 0.17	ß = -0.002<br>T = -0.64<br>p = 0.52	ß = -0.000<br>T = 0.11<br>p = 0.90
Movie<br>***R^2 = 0.49***<br>***df = 621***	**ß = -0.06**<br>**T = -23.76**<br>**p < 0.001**<br>**η^2 = 0.48**	**ß = -0.016**<br>**T = -6.72**<br>**p < 0.001**<br>**η^2 = 0.07**	ß = 0.001<br>T = 0.29<br>p = 0.77	ß = -0.003<br>T = -1.13<br>p = 0.26	ß = -0.002<br>T = -0.69<br>p = 0.49
SMT<br>***R^2 = 0.41***<br>***df = 621***	**ß = -0.05**<br>**T = -20.07**<br>**p < 0.001**<br>**η^2 = 0.39**	**ß = -0.015**<br>**T = -6.16**<br>**p < 0.001**<br>**η^2 = 0.057**	ß = 0.00<br>T = 0.08<br>p = 0.93	ß = -0.001<br>T = -0.35<br>p = 0.73	ß = 0.00<br>T = 0.99<br>p = 0.32

Supplementary Table 10 *Predicting SyS from Age in full sample (N=627) using Craddock parcellation.* Statistics from linear regression models predicting SyS from sex, a second-order polynomial expansion of age and their interactions. The terms age^1 and age^2 refer to the linear and quadratic effects of age respectively. Significant effects are shown in **bold**.

Task	Terms					
	SyS	SyS:age^1	SyS:age^2	SyS:Sex	Sex:age^1	Sex:age^2
Rest<br>***R^2 = 0.48***<br>***df = 615***	**ß = 0.48**<br>**T = 3.82**<br>**p < 0.001**<br>**η^2 = 0.02**	ß = -0.10<br>T = -0.82<br>p = 0.41	ß = -0.08<br>T = -0.68<br>p = 0.49	ß = -0.20<br>T = -1.57<br>p = 0.12	ß = -0.07<br>T = -0.55<br>p = 0.58	ß = -0.08<br>T = -0.68<br>p = 0.49
Movie<br>***R^2 = 0.48***<br>***df = 615***	**ß = 0.70**<br>**T = 4.62**<br>**p < 0.001**<br>**η^2 = 0.03**	ß = -0.18<br>T = -1.13<br>p = 0.26	ß = -0.08<br>T = -0.67<br>p = 0.50	ß = -0.11<br>T = -0.72<br>p = 0.47	ß = -0.06<br>T = -0.40<br>p = 0.69	ß = -0.02<br>T = -0.17<br>p = 0.87
SMT<br>***R^2 = 0.48***<br>***df = 615***	**ß = 0.43**<br>**T = 2.73**<br>**p = 0.006**<br>**η^2 = 0.02**	ß = 0.05<br>T = 0.32<br>p = 0.75	ß = -0.22<br>T = -1.75<br>p = 0.08	ß = -0.23<br>T = -1.49<br>p = 0.14	ß = -0.10<br>T = -0.69<br>p = 0.49	ß = -0.12<br>T = -0.96<br>p = 0.34

Supplementary Table 11 *Predicting Fluid Intelligence from SyS in full sample (N=627) using Craddock parcellation*. Statistics from linear models predicting Fluid Intelligence from SyS, after adjusting for sex, second-order effects of age and their interaction.

Task	Terms					
	SyS	SyS:age^1	SyS:age^2	SyS:Sex	Sex:age^1	Sex:age^2
Rest<br>***R^2 = 0.16***<br>***df = 615***	**ß = 0.68**<br>**T = 2.55**<br>**p = 0.01**<br>**η^2 = 0.004**	ß = -0.44<br>T = -1.61<br>p = 0.10	ß = 0.03<br>T = 0.11<br>p = 0.91	ß = -0.13<br>T = -0.50<br>p = 0.62	ß = -0.13<br>T = -0.50<br>p = 0.62	ß = 0.17<br>T = 0.74<br>p = 0.46
Movie<br>***R^2 = 0.16***<br>***df = 615***	**ß = 0.65**<br>**T = 2.01**<br>**p = 0.045**<br>**η^2 = 0.003**	**ß = -0.66**<br>**T = -1.98**<br>**p = 0.047**	ß = -0.09<br>T = -0.37<br>p = 0.71	ß = -0.25<br>T = -0.79<br>p = 0.43	ß = -0.41<br>T = -1.24<br>p = 0.21	ß = 0.35<br>T = 1.26<br>p = 0.20
SMT<br>***R^2 = 0.16***<br>***df = 615***	ß = 0.42<br>T = 1.27<br>p = 0.20<br>η^2 = 0.0003	ß = -0.68<br>T = -1.89<br>p = 0.059	ß = -0.05<br>T = -0.22<br>p = 0.82	ß = -0.39<br>T = -1.18<br>p = 0.24	ß = -0.42<br>T = -1.31<br>p = 0.19	ß = 0.22<br>T = 0.83<br>p = 0.41

Supplementary Table 12 *Predicting Episodic Memory from SyS in the full sample (N=627) using Craddock parcellation*. Statistics from linear models predicting Episodic Memory from SyS, after adjusting for sex, second-order effects of age and their interaction. We note the main effect of Movie SyS is not significant when performing robust regression.

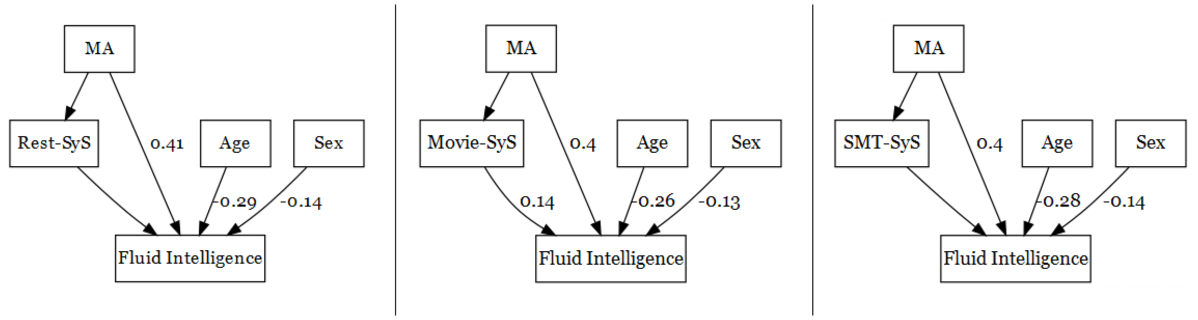

Supplementary Figure 20. *Mediation plot using Craddock Parcellation*. We did not evidence that SyS measure mediated the positive effect MA has on late life fluid intelligence. [**Rest**: Proportion mediated (PM) = 2.8%; Z = 1.13, p = 0.26; **Movie**: PM = 3.7%; Z = 1.26, p = 0.21; **SMT**: PM = 2.7%; Z = 1.14, p = 0.26].

Age and Cognition Effects (Gordon Atlas)

Here we used the Gordon et al., (2016) atlas to examine how SyS relates to age and cognition. The Gordon atlas defined ROIs based on boundary mapping approach applied to resting-state functional connectivity measure. This approach identifies regions by treating abrupt changes in voxel functional connectivity values as boundaries across cortical areas (see also (Wig et al., 2014)). Networks were identified based on community detection procedure (Infomap; (Rosvall & Bergstrom, 2008)); similar to how networks were identified in Power et al., (2011). This resulted in 13 networks. We again focused on associative networks, including: Cingulo-Opercular, Cingulo-Parietal, Default Mode Network, Dorsal Attention Network, Fronto-Parietal Network, Salience Network, Ventral Attention Network and Retrosplenial temporal Networks.

Figures and Tables for the Gordon atlas corresponding to those in the main paper (using Schaefer atlas) are shown below. The main results are replicated, particularly for fluid intelligence, although relationship with episodic memory is only significant in the Rest state.

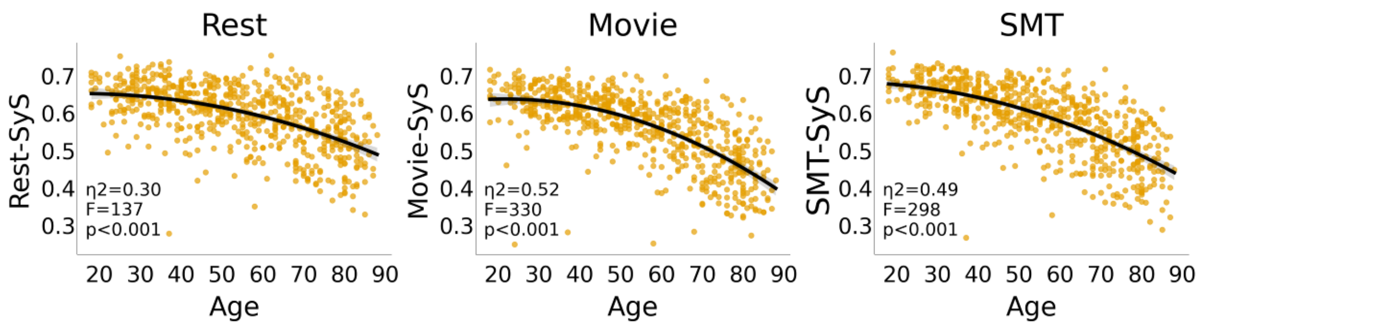

Supplementary Figure 21 *Predicting SyS from Age in full sample (N=627) using Gordon parcellation.* SyS decreases with age in each of the three brain states.

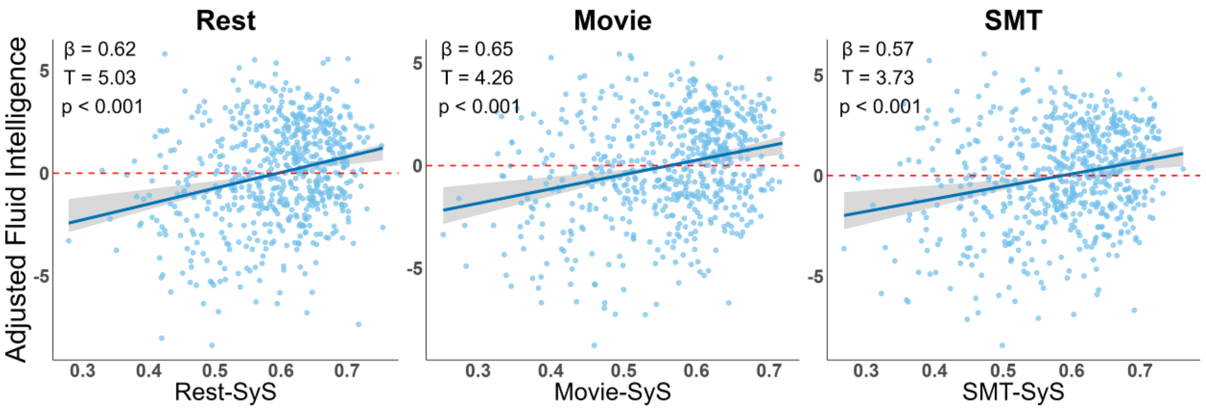

Supplementary Figure 22 *Predicting Fluid Intelligence from SyS in full sample (N=627) using Gordon Parcellation.*

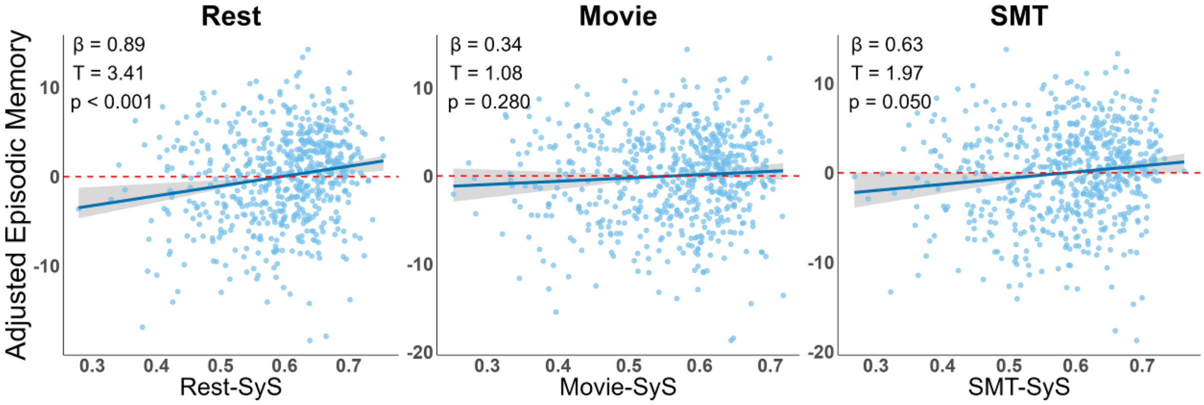

Supplementary Figure 23 *Predicting Episodic Memory from SyS in full sample (N=627) using Gordon Parcellation.*

Task	Polynomial Term				
	age¹	age²	Sex	age¹:Sex	age²:Sex
Rest<br>***R^2 = 0.30***<br>***df = 621***	**ß = -0.04**<br>**T = -16.20**<br>**p < 0.001**<br>**η^2 = 0.31**	**ß = -0.001**<br>**T = -3.48**<br>**p < 0.001**<br>**η^2 = 0.02**	ß = 0.003<br>T = 1.07<br>p = 0.28	ß = -0.00<br>T = 0.14<br>p = 0.89	ß = -0.001<br>T = -0.47<br>p = 0.64
Movie<br>***R^2 = 0.51***<br>***df = 621***	**ß = -0.06**<br>**T = -24.96**<br>**p < 0.001**<br>**η^2 = 0.52**	**ß = -0.02**<br>**T = -6.06**<br>**p < 0.001**<br>**η^2 = 0.06**	ß = 0.001<br>T = 0.47<br>p = 0.46	ß = -0.00<br>T = -0.02<br>p = 0.98	ß = -0.00<br>T = 0.25<br>p = 0.80
SMT<br>***R^2 = 0.49***<br>***df = 621***	**ß = -0.06**<br>**T = -24.06**<br>**p < 0.001**<br>**η^2 = 0.51**	**ß = -0.01**<br>**T = -4.17**<br>**p < 0.001**<br>**η^2 = 0.03**	ß = 0.00<br>T = 0.26<br>p = 0.79	ß = 0.001<br>T = 0.50<br>p = 0.61	ß = 0.001<br>T = 0.54<br>p = 0.59

Supplementary Table 13 *Predicting SyS from Age in full sample (N=627) using Gordon parcellation.* Statistics from linear regression models predicting SyS from sex, a second-order polynomial expansion of age and their interactions. The terms age¹ and age² refer to the linear and quadratic effects of age respectively. Significant effects are shown in **bold**.

Task	Terms					
	SyS	SyS:age^1	SyS:age^2	SyS:Sex	Sex:age^1	Sex:age^2
Rest<br>***R^2 = 0.49***<br>***df = 615***	**ß = 0.61**<br>**T = 5.03**<br>**p < 0.001**<br>**η^2 = 0.04**	ß = -0.08<br>T = -0.62<br>p = 0.54	ß = -0.09<br>T = -0.87<br>p = 0.38	ß = -0.23<br>T = -1.84<br>p = 0.06	ß = -0.10<br>T = -0.79<br>p = 0.42	ß = -0.12<br>T = -1.07<br>p = 0.28
Movie<br>***R^2 = 0.48***<br>***df = 612***	**ß = 0.65**<br>**T = 4.27**<br>**p < 0.001**<br>**η^2 = 0.03**	ß = -0.06<br>T = -0.40<br>p = 0.68	ß = -0.04<br>T = -0.36<br>p = 0.72	ß = -0.16<br>T = -1.05<br>p = 0.29	ß = -0.14<br>T = -0.88<br>p = 0.38	ß = -0.07<br>T = -0.53<br>p = 0.60
SMT<br>***R^2 = 0.48***<br>***df = 612***	**ß = 0.57**<br>**T = 3.73**<br>**p < 0.001**<br>**η^2 = 0.03**	ß = 0.12<br>T = 0.71<br>p = 0.48	ß = -0.16<br>T = -1.41<br>p = 0.16	ß = -0.25<br>T = -1.67<br>p = 0.09	ß = -0.14<br>T = -0.88<br>p = 0.38	ß = -0.15<br>T = -1.09<br>p = 0.28

Supplementary Table 14 *Predicting Fluid Intelligence from SyS in full sample (N=627) using Gordon parcellation*. Statistics from linear models predicting Fluid Intelligence from SyS, after adjusting for sex, second-order effects of age and their interaction. There was a significant 3 way interaction between Rest-SyS*age^1*Sex (β = -0.58, t = -2.2, p = 0.027)

Task	Terms					
	SyS	SyS:age^1	SyS:age^2	SyS:Sex	Sex:age^1	Sex:age^2
Rest<br>***R^2 = 0.17***<br>***df = 615***	**ß = 0.89**<br>**T = 3.41**<br>**p < 0.001**<br>**η^2 = 0.015**	ß = -0.30<br>T = -1.10<br>p = 0.27	ß = -0.18<br>T = -0.79<br>p = 0.43	ß = 0.03<br>T = 0.11<br>p = 0.91	ß = -0.11<br>T = -0.40<br>p = 0.68	ß = 0.19<br>T = 0.79<br>p = 0.43
Movie<br>***R^2 = 0.16***<br>***df = 612***	ß = 0.34<br>T = 1.08<br>p = 0.28<br>η^2 = 0.0003	ß = -0.47<br>T = -1.41<br>p = 0.15	ß = -0.09<br>T = -0.38<br>p = 0.70	ß = -0.41<br>T = -1.29<br>p = 0.19	ß = -0.55<br>T = -1.65<br>p = 0.09	ß = 0.38<br>T = 1.36<br>p = 0.17
SMT<br>***R^2 = 0.16***<br>***df = 613***	**ß = 0.63**<br>**T = 1.97**<br>**p = 0.049**<br>**η^2 = 0.003**	ß = -0.74<br>T = -2.15<br>p = 0.03	ß = -0.16<br>T = -0.66<br>p = 0.51	ß = -0.25<br>T = -0.77<br>p = 0.44	ß = -0.39<br>T = -1.13<br>p = 0.26	ß = 0.24<br>T = 0.81<br>p = 0.42

Supplementary Table 15 *Predicting Episodic Memory from SyS in the full sample (N=627) using Gordon parcellation*. Statistics from linear models predicting Episodic Memory from SyS, after adjusting for sex, second-order effects of age and their interaction. We there was a marginally significant 3-way interaction between Mov-SyS*Age^2*Sex (β = -0.91 $t_{(612)}$= -1.93 p = 0.053).

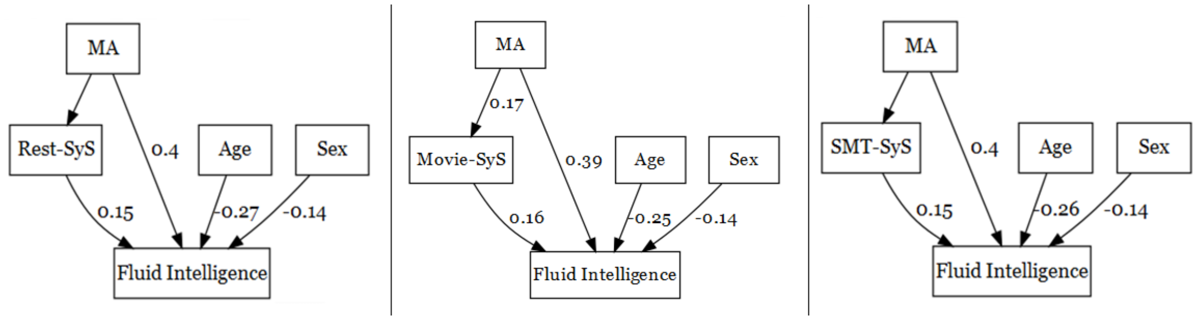

Supplementary Figure 24 *Mediation models with Gordon parcellation.* We did not evidence that SyS measure mediated the positive effect MA has on late life fluid intelligence. [**Rest**: Proportion mediated (PM) = 3.6%; Z = 1.23, p = 0.22; **Movie**: PM = 6.4%; Z = 1.74, p = 0.08; **SMT**: PM = 4.8%; Z = 1.47, p = 0.14]

Effect of different pre-processing options

There are many important pre-processing choices in estimating functional connectivity from fMRI. Using the effect of SyS on cognition (adjusting for age and sex) as our “test case”, we examined the robustness of this effect to other pre-processing options (for all three brain states and both cognitive measures). More specifically, we computed SyS after varying 4 different pre-processing options. Here we report measures computed from the Schaefer atlas. However, we note similar effects of pre-processing were observed when we used SyS measured computed with the Schaefer parcellation. Note this is not an exhaustive list of pre-processing options that can be varied; nor is it a formal comparison across pipelines (the latter would benefit from large datasets with repeated measures to measure the reliability of effects).

1) The first alternative option was not to include global signal regression (GSR), which was used in the original M. Chan et al. (2014) paper, but has been criticised by others (see fore review Murphy & Fox, 2017; Saad et al., 2012). Here we did not perform GSR, but still zeroed negative connections. We still found a positive association between SyS in the Movie and SMT states and fluid intelligence after accounting for age and sex effects (Supplementary Figure 25). The association between Rest-SyS and fluid intelligence did not reach significance after taking into consideration the effects of age and sex. We again did not observe an association between SyS and episodic memory in any of the brain states.

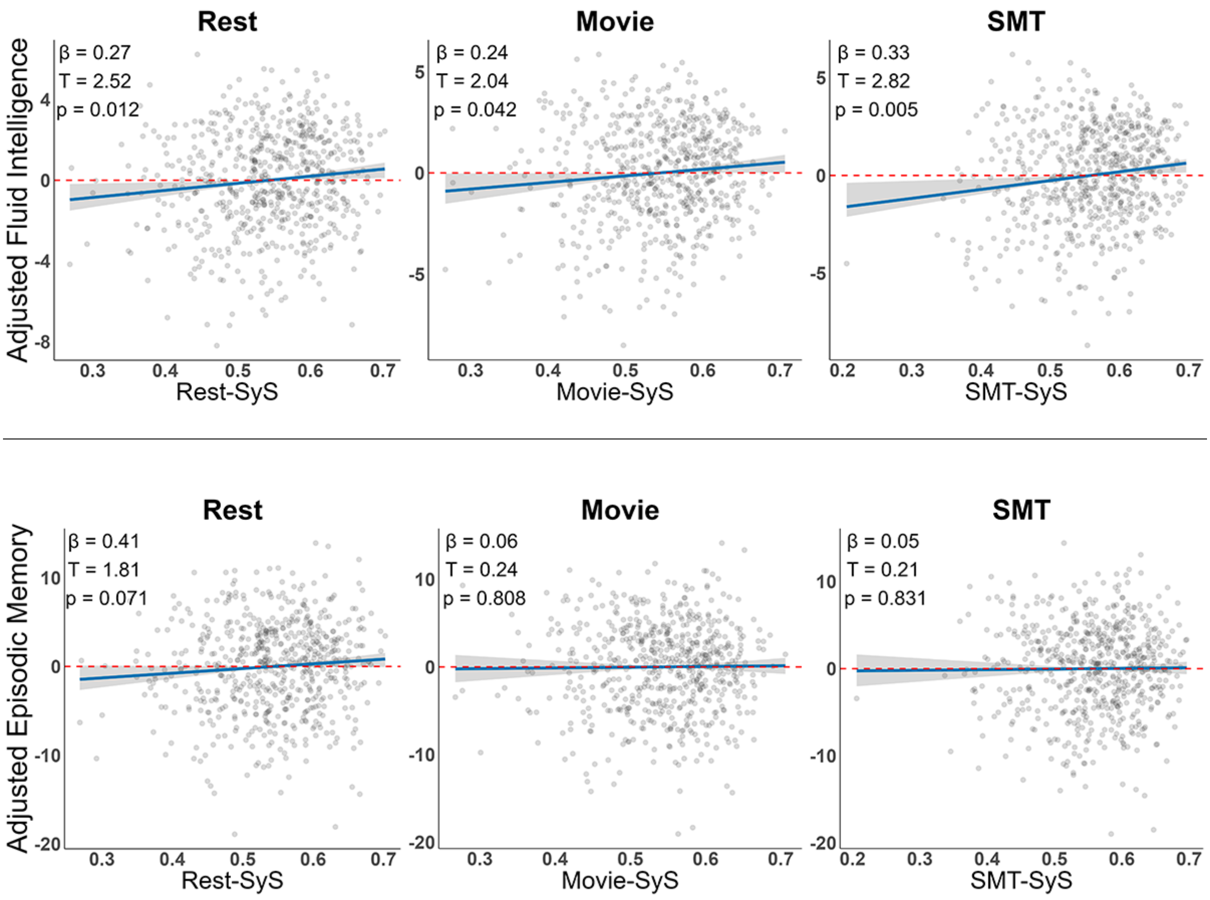

Supplementary Figure 25 *SyS and Cognition when SyS was computed without performing GSR.* Blue line indicates linear fit, and shaded area demonstrates 95% intervals for the line fit.

2) Continuing without GSR, we used mean regression (MR) of the average connectivity across all nodes, which we previously found effective at adjusting for vascular effects of ageing (Geerligs et al., 2017; Yan et al., 2013). We still excluded negative connections. We again find that SyS is positively associated with fluid intelligence, but not with episodic memory, across the three brain states (Supplementary Figure 26).

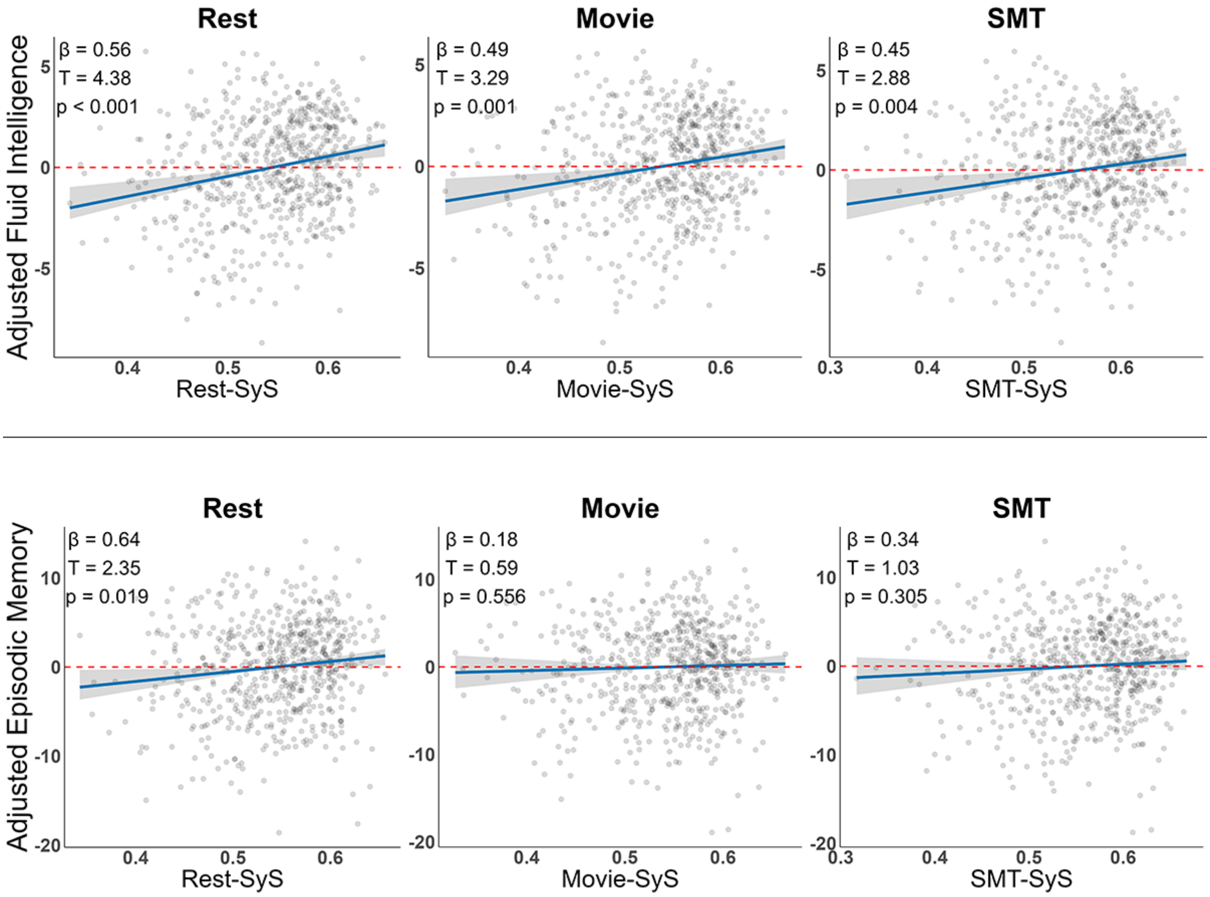

Supplementary Figure 26 *SyS and Cognition when SyS was computed without performing GSR but performing MR.* Blue line indicates linear fit, and shaded area demonstrates 95% intervals for the line fit.

3) Rather than using GSR or MR, we computed the edges using partial correlation. Partial correlation is thought to better capture direct association between ROIs, i.e., by accounting for indirect connectivity via intervening ROIs. We performed partial correlation using the FSLnets package (Smith et al., 2011), which uses the regularised inverse covariance matrix (ICOV) to estimate direct pairwise connectivity across regions. Any association between SyS and cognition, after controlling for age and sex, was no longer significant when using partial correlation (Supplementary Figure 27).

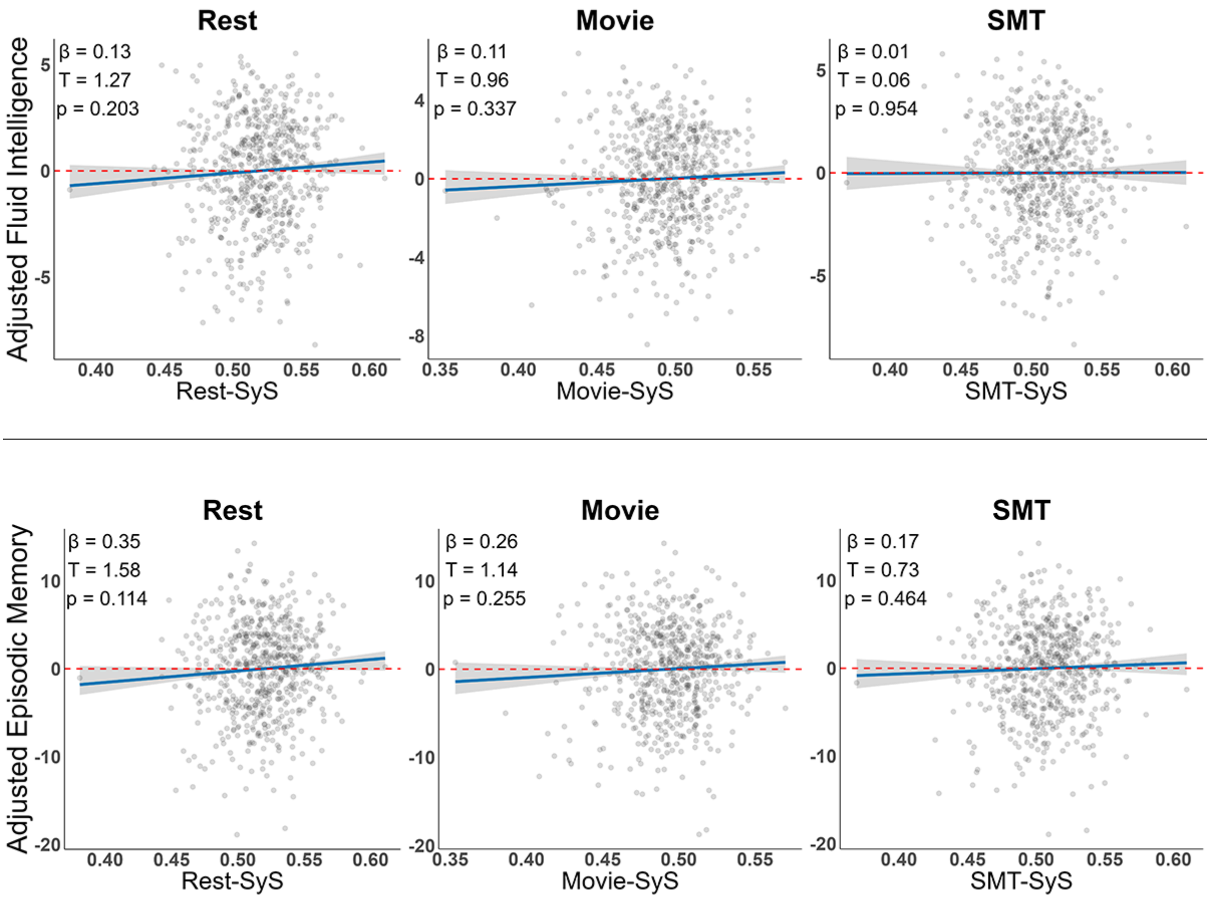

Supplementary Figure 27 *SyS and Cognition. SyS was computed without performing GSR, but the pairwise connectivity between regions was computed using partial correlation rather than full correlation.* Blue line indicates linear fit, and shaded area demonstrates 95% intervals for the line fit.

4) Finally, we continued without GSR or MR, but this time did not exclude negative connections (i.e., included all edges in calculating SyS). This removed the association between SyS and cognition (Supplementary Figure 28), suggesting that some thresholding is necessary, e.g., to maintain only positive correlations.

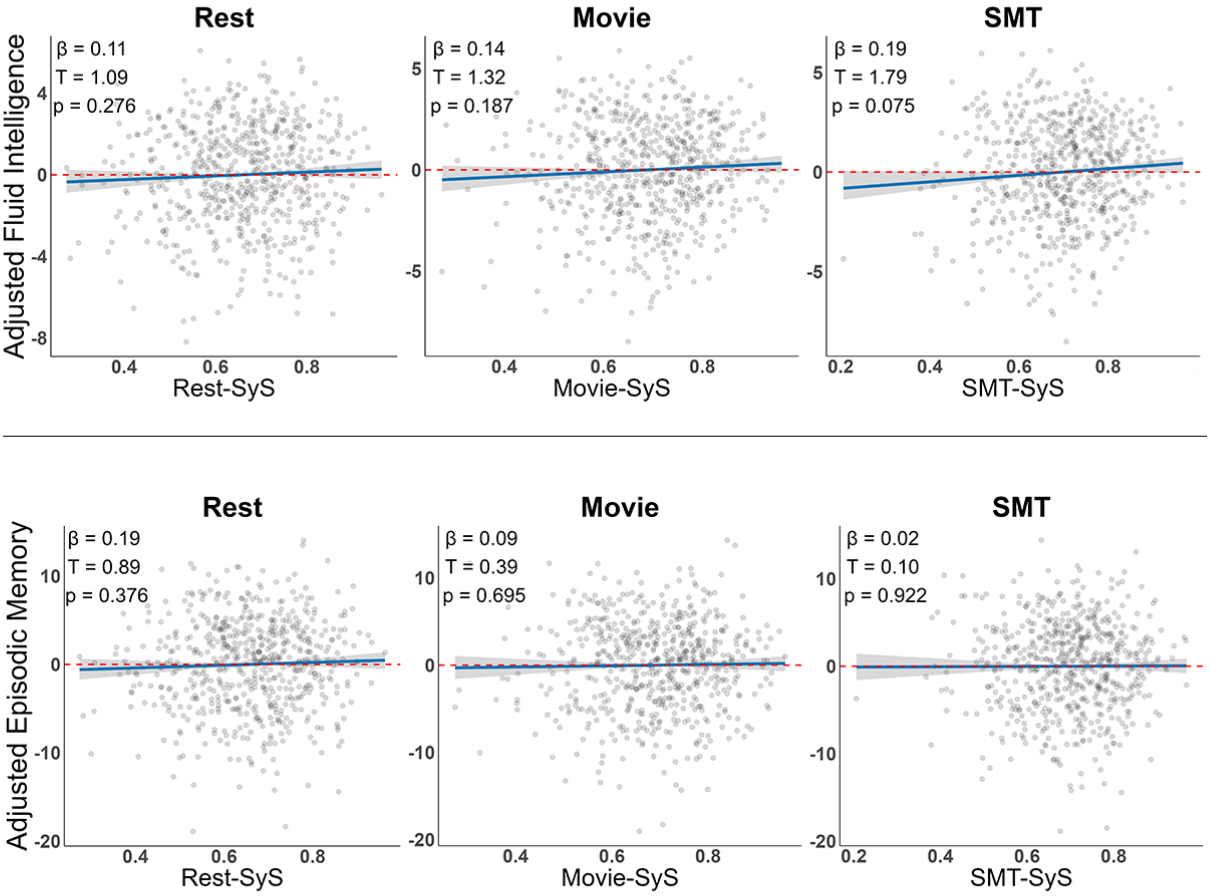

Supplementary Figure 28. *SyS and Cognition when SyS was computed without performing GSR with both positive and negative edges.* Blue line indicates linear fit, and shaded area demonstrates 95% intervals for the line fit.

References

Chan, M. Y., Park, D. C., Savalia, N. K., Petersen, S. E., & Wig, G. S. (2014). Decreased segregation of brain systems across the healthy adult lifespan. *Proceedings of the National Academy of Sciences*, *111*(46), E4997–E5006. https://doi.org/10.1073/pnas.1415122111

Craddock, R. C., James, G. A., Holtzheimer III, P. E., Hu, X. P., & Mayberg, H. S. (2012). A whole brain fMRI atlas generated via spatially constrained spectral clustering. *Human Brain Mapping*, *33*(8), 1914–1928.

Geerligs, L., Rubinov, M., & Henson, R. N. (2015). State and trait components of functional connectivity: individual differences vary with mental state. *Journal of Neuroscience*, *35*(41), 13949–13961. https://doi.org/10.1523/JNEUROSCI.1324-15.2015

Geerligs, L., Tsvetanov, K. A., & Henson, R. N. (2017). Challenges in measuring individual differences in functional connectivity using fMRI: the case of healthy aging. *Human Brain Mapping*, *38*(8), 4125–4156. https://doi.org/10.1002/hbm.23653

Gordon, E. M., Laumann, T. O., Adeyemo, B., Huckins, J. F., Kelley, W. M., & Petersen, S. E. (2016). Generation and evaluation of a cortical area parcellation from resting-state correlations. *Cerebral Cortex*, *26*(1), 288–303. https://doi.org/10.1093/cercor/bhu239

Lancichinetti, A., & Fortunato, S. (2012). Consensus clustering in complex networks. *Scientific Reports*, *2*(1), 1–7.

Murphy, K., & Fox, M. D. (2017). Towards a consensus regarding global signal regression for resting state functional connectivity MRI. *NeuroImage*, *154*, 169–173. https://doi.org/https://doi.org/10.1016/j.neuroimage.2016.11.052

Power, J. D., Cohen, A. L., Nelson, S. M., Wig, G. S., Barnes, K. A., Church, J. A., Vogel, A. C., Laumann, T. O., Miezin, F. M., & Schlaggar, B. L. (2011). Functional network organization of the human brain. *Neuron*, *72*(4), 665–678.

Rosvall, M., & Bergstrom, C. T. (2008). Maps of random walks on complex networks reveal community structure. *Proceedings of the National Academy of Sciences*, *105*(4), 1118–1123.

Saad, Z. S., Gotts, S. J., Murphy, K., Chen, G., Jo, H. J., Martin, A., & Cox, R. W. (2012). Trouble at rest: how correlation patterns and group differences become distorted after global signal regression. *Brain Connectivity*, *2*(1), 25–32. https://doi.org/10.1089/brain.2012.0080

Smith, S. M., Miller, K. L., Salimi-Khorshidi, G., Webster, M., Beckmann, C. F., Nichols, T. E., Ramsey, J. D., & Woolrich, M. W. (2011). Network modelling methods for FMRI. *NeuroImage*, *54*(2), 875–891. https://doi.org/https://doi.org/10.1016/j.neuroimage.2010.08.063

Wig, G. S., Laumann, T. O., & Petersen, S. E. (2014). An approach for parcellating human cortical areas using resting-state correlations. *Neuroimage*, *93*, 276–291.

Yan, C.-G., Craddock, R. C., Zuo, X.-N., Zang, Y.-F., & Milham, M. P. (2013). Standardizing the intrinsic brain: Towards robust measurement of inter-individual variation in 1000 functional connectomes. *NeuroImage*, *80*, 246–262. https://doi.org/https://doi.org/10.1016/j.neuroimage.2013.04.081
